# Characterization of bacteria colonizing the mucosal layer of the gastrointestinal tract of Atlantic salmon farmed in a warm water region

**DOI:** 10.3389/fmicb.2025.1564052

**Published:** 2025-07-23

**Authors:** Chantelle E. Reid, Richard S. Taylor, Andrew Bissett, Barbara F. Nowak, John P. Bowman

**Affiliations:** ^1^Tasmanian Institute of Agriculture, University of Tasmania, Hobart, TAS, Australia; ^2^CSIRO Agriculture and Food, Battery Point, Hobart, Tasmania, Australia; ^3^Institute of Marine and Antarctic Studies, University of Tasmania, Hobart, TAS, Australia

**Keywords:** Atlantic salmon, temperature, *Aliivibrio*, *Vibrio*, *Photobacterium*, colonization

## Abstract

Atlantic salmon (*Salmo salar*) farmed in seawater in Tasmania (*lutruwita*) can experience temperatures close to their thermotolerance limit during summer. Gut microbiome data from eight successive annual surveys and a specific survey of GI tract mucosa and digesta bacterial cross-sectional distributions indicated that members of the genus *Aliivibrio, Vibrio*, and an unclassified Mycoplasmoidaceae are the main colonizers of the gut mucosal layer in Tasmanian farmed salmon. Peak abundance levels were reached 7–8 months after the transfer of smolt to sea cages from hatcheries. This corresponds to late summer, with the transfer of hatchery smolt occurring in winter. Salmon *Aliivibrio* isolates comprise three novel non-bioluminescent species. Along with other *Aliivibrio* species, these species have genes in common required for host colonization and biofilm formation, and also include species- and strain-level dependent features. Two of the novel *Aliivibrio* species surprisingly possessed genes for cytolethal distending toxin, while the more predominant species lacked any known virulence genes. The overall observations suggest a restricted group of species actively colonizes the mucosal layer of Atlantic salmon farmed in Tasmania, and that this process is strongly influenced by environmental temperature.

## Introduction

In Tasmania (Australia), also referred to as *lutruwita*, the Atlantic salmon industry is a major economic activity and relies on sea cage farming. The industry operates at the high end of the temperature scale due to summer sea surface temperatures averaging 15–20°C, while Atlantic salmon grow optimally at 13–16°C (Calado et al., 2021). There are concerns that sea surface temperatures in the southeast of Tasmania will increase over the long term due to the ongoing topicalization of the Eastern Australian Current (Oliver et al., 2017). An unprecedented 117-day “heat wave” during the summer of 2015–2016, when surface (5 m depth) temperatures reached almost 23°C, caused significant negative effects on the health of local farmed salmon and their downstream marketability (Wade et al., 2019). Increased warming is predicted to have potentially detrimental effects (Grünenwald et al., 2019; Hudson et al., 2022; Meng et al., 2022) on the Atlantic salmon industry.

Gut microbiome studies on Atlantic salmon and other related marine farmed finfish have been performed in various contexts. This research aims to understand the effects of environmental conditions, compare life stages, establish health status indicators, and connect this data to farm performance and feed formulations (Egerton et al., 2018). One of the challenges in these studies has been understanding the main players in the microbiome, how this varies between growing regions, and what the microbiome indicates for Atlantic salmon health and performance. The overall commercial system is complex and only approximates the anadromous lifestyle of Atlantic salmon. Typically, juvenile salmon are reared in freshwater hatcheries until they reach the smolt life stage (Li et al., 2023). By increasing light exposure and temperature, maturation and associated osmoregulation (Ytrestøyl et al., 2023) are stimulated, mimicking the natural spring–summer transition in the physiology of wild salmon. This artificial stimulus enables the smolt to rapidly adapt to seawater conditions, after which they are reared in marine farms (Hvas et al., 2021). The gut microbiota of Atlantic salmon in marine farms has been observed to change unidirectionally (Zarkasi et al., 2014; Lorgen-Ritchie et al., 2021; Wang et al., 2021). These changes have been associated with fish age, feeding rates, and weight gain in Chinook salmon (Zhao et al., 2020).

Studies to date clearly indicate that bacteria are predominant and establish populations in the gastrointestinal mucosal layer and digesta of Atlantic salmon. The main autochthonous adherent taxa, their distribution within the gut (from pyloric caeca to distal gut), and differences among aquaculture regions are now being established at high resolution (Vera-Ponce de León et al., 2024). A major autochthonous species present in wild Atlantic salmon has been described as “*Candidatus* Mycoplasma salmoninarum” (Rasmussen et al., 2023), a member of the genus *Malacoplasma* in the family Mycoplasmoidaceae. This species is found in abundance in the gut of wild post-smolt (Llewellyn et al., 2016) as well as in salmon farmed in sea pens worldwide. Abundance ranges from being completely dominant to only a sporadic presence (Zarkasi et al., 2014; Dehler et al., 2017; Fogarty et al., 2019; Bozzi D Rasmussen et al., 2021; Huyben et al., 2020; Wang et al., 2021; Brealey et al., 2022). In the Tasmanian salmon-growing region (147°E 43°S), Vibrionaceae (*Aliivibrio, Vibrio, Photobacterium*) occur at elevated levels in adult fish (Reid et al., 2024). Vibrionaceae are also common in ocean-dwelling wild salmon and in other farming regions (Wang et al., 2020, 2021, 2023; Lorgen-Ritchie et al., 2021; Llewellyn et al., 2016; Brealey et al., 2022; Huyben et al., 2020; Godoy et al., 2015; Dhanasiri et al., 2023; Vera-Ponce de León et al., 2024). Similar colonization patterns also occur in other anadromous, farmed salmonids such as rainbow trout (Rimoldi et al., 2021) and Chinook salmon (Ciric et al., 2019; Zhao et al., 2020; Ziab et al., 2023). For example, in Chinook salmon, which are extensively farmed in New Zealand, *Aliivibrio* and *Photobacterium* predominate in fish from marine farms, while *Aliivibrio* does not occur in fish reared in alpine freshwater systems (Zhao et al., 2020).

Certain members of Vibrionaceae cause disease in Atlantic salmon, and the most problematic have been managed through vaccination (Skåne et al., 2022). More avirulent species may dominate in fish that exhibit signs of dysbiosis, including reduced voluntary feeding and the presence of fecal casts. Reduced feeding can lead to anorexic-like states in a portion of fish (Hevrøy et al., 2012). Casts represent sloughed intestinal mucosa that appears as white to yellow fecal matter (Reid et al., 2024). Both phenomena occur in fish that are thermally stressed, though whether Vibrionaceae are directly involved in dysbiotic symptoms has yet to be demonstrated. *Aliivibrio* species are of particular interest, not only due to their potential to cause disease but also because of their probiotic capacity (Klakegg et al., 2020). These characteristics suggest an inherent ability for host colonization. Significant knowledge of host interactions gained from the model species *Aliivibrio fischeri* (Visick et al., 2021) provides a foundation for functional comparisons among different *Aliivibrio* species colonizing fish GI tracts.

Due to the predominance of Vibrionaceae in marine-farmed fish, understanding their biology is important. This includes capabilities related to persistent colonization, growth *in vivo*, response to water temperatures, and connection to dysbiosis. In this study, we examined the colonization of Atlantic salmon reared in marine pens located in the D'Entrecasteaux Channel region of Tasmania. Unlike previous studies, we performed an analysis of gut microbiome samples collected over multiple years (2010–2018) to determine colonization patterns. Secondly, we compared the distribution of bacteria throughout the GI tract by examining different gut sections collected from two separate fish cohorts in summer and winter. This was done to more definitively answer the question of which bacteria show proliferation in the gut mucosa vs. simply passive transit via feed or water. To understand the traits of actively colonizing bacteria in the gut of Atlantic salmon, we also isolated strains from salmon digesta samples and sequenced their genomes. From this, we examined their taxonomy and the relevant genes the strains possess that enable host colonization. Finally, we wanted to know if gut colonizers possess virulence genes, maintaining a focus on the relevance of strain colonization as a facet of the health, welfare, and farm performance of Atlantic salmon, especially in commercial settings. From these studies, we can show that Atlantic salmon is actively colonized by distinct species with a strong capability for growth in the mucosal layer of the Atlantic salmon GI tract.

## Materials and methods

### Sea pen sampling

For sea-pen-derived fish, data in this study included different surveys performed between 2010 and 2018 in the southeast of Tasmania (Table 1, Supplementary Figure S1). Four of these surveys have been previously published (Zarkasi et al., 2014, 2016; D'Agnese et al., 2023; Reid et al., 2024). New data are from other locations in the same region (Table 1, Supplementary Figure S1). In each studied cohort, smolt were put to sea over winter (July–August). In one survey, samples were also collected between 0 and 3 weeks prior to transfer from two hatcheries.

**Table 1:** Experimental datasets utilized in the study, including design, data sources, and related information.

**Experiment type**	**Location of sampling**	**16S rRNA primers**	**Sequencing method**	**Time period samples collected**	**Number of samples per time point**	**Fish weigh range (kg)**	**Average reads per sample (DADA2)^a^**	**References**	**NCBI sequence read accession code**	**Animal ethics approval no**.
Farm-based	Meads Creek, Port Esperance (1)	V3–V5	Pyrosequencing	Aug 2010–Apr 2011	20, 49, 50, 50, 50	0.4–4.1	3,862	Zarkasi et al., 2016	PRJNA1021267	DPIPWE 30/2009-10
Farm-based	Robert's Point lease, D'Entre- casteaux Channel (2)	V3–V5	Pyrosequencing	Jul 2011–Aug 2012	6, 12, 12, 11, 12, 11, 10, 12, 12	0.5–1.8	4,009	Zarkasi et al., 2014	PRJNA1021267	UTAS A0012001
Farm-based	North West Bay (3)	V1–V3	Illumina MiSeq	Nov 2013–Jun 2014	9, 8, 11	Not available	71,700	This study	PRJNA1021367	UTAS A0013971
Farm-based	Red Cliffs, D'Entrecasteaux Channel (4)	V1–V3	Illumina MiSeq	Jan 2014–Jun 2014	64, 92, 49, 76	1.2–2.2	55,255	D'Agnese et al., 2023	PRJNA979964	UTAS A0013971
Farm-based	North West Bay (3)	V1–V3	Illumina MiSeq	Jan 2015–Apr 2015	25, 14, 23	Not available	28,029	This study	PRJNA1021684	UTAS A0013971
Farm-based	Soldier's Point, D'Entrecasteaux Channel (5)	V3–V5	Illumina MiSeq	Dec 2015–May 2016	16, 30, 21, 11	Not available Not available	26,127	This study	PRJNA1021687	UTAS A0015452
Farm-based	Red Cliffs lease, Sheppards lease, D'Entrecasteaux Channel (4, 6)	V1–V3	Illumina MiSeq	Jan 2018, Jul 2018^b^	182, 196	2.9	15,413	Reid et al., 2024	PRJNA1025921, PRJNA1026149	UTAS A0016307 A0016588, A0017010
Hatcheries/farms	Feed pellets, water (hatchery), water (farm-site), smolt, farms—Robert's Point Lease, Sheppards leases, D'Entre-casteaux Channel (4, 6)	V1–V3	Illumina MiSeq	May 2017–May 2018	40, 28, 12, 90, 81	0.05–0.15 (smolt), 0.1–0.9 (farm)	6,041, 12,315, 9,788, 16,694, 9,990	This study	PRJNA1025868	UTAS A0016307 A0016588, A0017010

a Samples with < 500 reads were not included in the study.

b Samples were collected from fore-, mid-, and hindgut digesta and mucosa, as well as by fecal stripping in the 24 h period after fish were fed (Reid et al., 2024).

### Digesta, feed pellet, and water sampling

Groups of Atlantic salmon were captured using a large seine net, which was shallowed to crowd the fish to ensure random mixing. Fish were sampled randomly with a dip-net. The captured fish were transferred into 300 L aerated tubs containing 17 ppm Aqui-S. For fecal stripping, the ventral surfaces of anesthetized fish were wiped with ethanol to minimize contamination, and samples were obtained by gently pinching and massaging the fish from the midline above the pelvic fins down toward the vent. Initial samples stripped from each fish were visually scored using the fecal consistency scoring method of Zarkasi et al. (2016). Following fecal scoring, 200–300 mg of digesta was aseptically collected and placed into a sterile 2 mL tube. The samples were then snap-frozen in a dewar containing liquid nitrogen and transported back to the laboratory, where they were stored at −80°C until DNA was extracted. All sampling from either hatcheries or farms was performed under University of Tasmania Animal Ethics Permits, as listed in Table 1. Considerable steps were taken to minimize cross-contamination by using sterile surgical sheets and disinfecting dissecting equipment by immersion in 95% (v/v) ethanol, followed by a 10% (v/v) sodium hypochlorite solution for DNA decontamination (Nilsson et al., 2022). Water samples were collected at all sampling events by submerging and filling autoclaved 2 L glass laboratory bottles with either inlet water or directly from tanks, representing microbes in the surrounding environment. This water was then filtered through 0.22 μm Sterivex (Merck Millipore) filter units using a peristaltic pump, and the filters were frozen at −80°C for further processing. The pump was sterilized between samples by pumping through 0.1% NaClO, then flushing through, and any remaining NaClO solution was rinsed away using sterile distilled water. Due to the influence of DNA from feed pellets (Karlsen et al., 2022), feed pellet samples were collected from automatic feeders and transferred directly to sterile zip lock bags. All samples were kept frozen at −80°C until processed.

### DNA extraction of samples and sequencing

Total genomic DNA from digesta and feed pellets (0.25 g frozen weights) was extracted following the manufacturer's specifications using the QIAamp DNA Stool Kit (Qiagen Cat. No. 51604). To ensure full bacterial lysis, the lysis incubation temperature was increased to 95°C, and the incubation time was extended to 10 min for all samples. Isolation of genomic DNA from Sterivex filtered water samples was processed using the DNeasy PowerWater Sterivex Kit (Qiagen Cat No. 14600-50-NF) following the manufacturer's specifications. The concentration of extracted DNA was determined using a NanoDrop 8000 spectrophotometer (Thermo Fisher Scientific).

### Sequence processing and classification

Sequencing was performed at the Ramaciotti Center for Genomics, University of NSW, Sydney, Australia, using the Illumina MiSeq platform. For this, 16S rRNA genes were amplified using V1/27F 5′-AGA GTT TGA TYM TGG CTC AG-3′ (Lane, 1991) and V3/519R 5′-GWA TTA CCG CGG CKG CTG-3′ (Turner et al., 1999) primers. The Illumina pipeline automatically trimmed adaptors from the 3′ prime end of sequences. All sequence data (Table 1) were processed using DADA2 (Callahan et al., 2016) in Galaxy Australia to create ASV tables. The ASVs were then classified using megaBLAST v. 2.2.26+ against the GreenGenes2 database (McDonald et al., 2024) in SEED2 (Větrovský et al., 2018).

### Core microbiome and relative abundance of bacterial genera over time

Core microbiome membership was considered based on a combination of prevalence in samples and abundance. For this, the sample read data were pooled by sampling period or type, including samples from hatchery water, farm-site water, feed pellets, the first winter, spring, summer, autumn, and the second winter. The ASV abundances at the genus level were then subsampled to the smallest group (using generateSubsampledMatrix in subseq v. 1.38.0, Robinson and Storey, 2024) in R Studio v. 4.5.0. From this, the proportional representation of the most abundant genera was arrayed in a bubble matrix (prepared in Excel v. 2501). CoDaPack v. 2.03.06 (Comas and Thió Fernández de Henestrosa, 2011) was used to convert the non-subsampled data to centered log ratios (CLR) for normalization and to account for data compositionality. Designated core microbiome members of Atlantic salmon hindgut samples were then compared based on the months since transfer to marine farms. The results were displayed as bee swarm plots generated using BoxPlotR (January 17, 2021 version; Spitzer et al., 2014).

### Enumeration, isolation, and 16S rRNA gene analysis of salmon gut bacteria

Atlantic salmon digesta was suspended and serially diluted in a sterile 3% (w/v) sea salt solution (Instant Ocean, Aquarium Systems) and plated onto marine agar (MA−0.5% w/v Bacto-peptone, 0.2% w/v yeast extract, 3.5% w/v seawater salts, 1.5% w/v agar), Thiosulfate Citrate Bile Sucrose agar (TCBS, Thermo Fisher Scientific, Scoresby, VIC, Australia), and de Man–Rogosa–Sharpe agar (MRS, Thermo Fisher Scientific). Plates were incubated at 25°C for up to 7 d. Counts were calculated as colony-forming units (CFU) per g of feces (wet weight). For various salmon samples (obtained between 2015/2016 and 2017/2018), bacterial isolates were picked from colonies on MA agar plates and then purified. Pure cultures were cryopreserved in marine broth that included 30% (v/v) glycerol and stored at −80°C. DNA was extracted from each strain using the Ultraclean Microbial DNA Extraction kit (Qiagen). Isolates were identified using 16S rRNA gene sequence analysis. Amplification of the 16S rRNA gene used 10 pmol/μL 27F and 1492R 5′-GGT TAC CTT GTT ACG ACT T-3′ (Lane, 1991) primers. PCR utilized MyTaq PCR reagent (Bioline) and included 1 mL (20–50 ng) of template DNA. The thermocycler (Peltier PTC200, MJ Research) conditions were 95°C for 1 min (1 cycle); 95°C for 30 s, 50°C for 30 s, 72°C for 30 s (34 cycles); 72°C for 10 min (1 cycle); 10°C soak. PCR products were purified and then sequenced using BigDye chemistry on an AB3730xl (Applied Biosystems). Sequence end regions were trimmed to remove poorly resolved base calls and aligned to reference sequences downloaded from the NCBI database. Phylogenetic trees were generated using maximum likelihood (PhyML) and neighbor-joining (BioNJ) in phylogeny.fr (Lemoine et al., 2019). Trees were visualized using the Interactive Tree of Life (Letunic and Bork, 2021).

### Growth analyses and biofilm formation ability

To assess responses to media salinity, the cultures were grown in marine broth in which sea salts were replaced by different concentrations of NaCl (0–10% NaCl). Bile salt tolerance was assessed similarly, using marine broth supplemented with Oxoid bile salts no. 3 (0–5%, Thermo Fisher Scientific). The ability of isolates to form biofilms was evaluated using the crystal violet assay in 96-well polystyrene trays and on acid-washed glass surfaces, as previously described (Hussa et al., 2008; Hansen et al., 2014). The motility of cultures (incubated in marine broth for 40–72 h at 20°C) was observed under phase contrast microscopy using wet mounts. For the determination of biokinetic growth responses, representative isolates were grown in marine broth at 25°C for 48 h. Inoculum was transferred to fresh media and grown again at 25°C for 24 h. Cultures were then diluted to 10^4^-10^5^ CFU/mL in fresh marine broth and aliquoted into custom-designed L-shaped 15-mL test tubes. The L-shaped tubes were placed into a custom-built aluminum block temperature gradient incubator (Terratec, Kingston, Tasmania) and incubated at temperatures ranging from 2 to 40°C. Optical density readings were taken using a Spectronic 200 (ThermoScientific) until no change in absorbance values was evident. Growth rates were determined using the logistic D model of Baranyi and Roberts (1994), with cardinal growth temperatures estimated by fitting the data with the Ratkowsky model (Ratkowsky et al., 1983) using ratkowsky_1983.R (Padfield et al., 2021).

### Genome assembly and annotation

Genome sequence data were obtained using either the Illumina HiSeq or the NovaSeq 6000 platforms, generating either 200 or 250 bp paired-end sequences. Fastq files were uploaded to the Galaxy Australia cloud server, and assemblies were generated using either SPAdes (various versions, Prjibelski et al., 2020) or Unicycler v. 0.5.1-galaxy0 (Wick et al., 2016). Default parameters were used for the SPAdes assembly process, while the bold bridging mode was used for Unicycler assembly analysis. Genome assemblies were then assessed using CHECKM2 (Chklovski et al., 2023). Genomes were annotated using the PANNZER2 server (Törönen and Holm, 2022), which makes predictions using ARGOT (Lavezzo et al., 2016). Specific 16S rRNA gene variant regions were also detected by manual inspection of joined paired-end read data for selected *Aliivibrio* genomes (strains LMG23869T, A2, A9, A32, S4MY1, and S3MY1), based on DADA2 ASV findings.

### Genome-based taxonomy

Genomes were compared using ortho-ANI (Lee et al., 2016) and GGDC 3.0 model 2 (Meier-Kolthoff et al., 2013) to discern putative species-level groups, assuming that 95% and 70% demarcate approximate boundaries for species, respectively. The V1–V3 regions of 16S rRNA gene sequences from available genomes of other *Aliivibrio* species, as well as placeholders defined by Klemetsen et al. (2021) and in GTDB (Parks et al., 2022), as well as abundant ASVs from the DADA2 analysis, were compared. 16S rRNA gene trees with bootstraps were generated utilizing maximum likelihood model inference in IQTREE (Minh et al., 2020) with 1,000 bootstraps in ultrafast mode. Since ANI and GGDC-based trees rely on fixed similarity values, they were analyzed using the BioNJ algorithm (Gascuel, 1997) in phylogeny.fr, with visualization in ITOL.

### Differential abundance analysis between pairs of gut digesta and mucosa

Comparisons of relative abundances of bacterial taxa enriched in digesta and mucosal samples from distinct parts of the gastrointestinal tract of Atlantic salmon were performed. For this analysis, the digesta and mucosal samples compared were from two different sets of 27 fish sampled in summer and winter (Reid et al., 2024, Table 1). The sampling approach enabled comparisons of different sections of the gut, including the foregut (pyloric caecum), midgut, and hindgut within the same fish. Paired comparisons were made on samples from the hindgut and midgut digesta and mucosal samples in relation to equivalent foregut samples. This was done to capture changes occurring across the sampled gut length. It was assumed that proliferating bacteria would become more abundant progressing from the foregut to the hindgut, where bacterial populations are highest. Similarly, it was assumed that feed-derived DNA would undergo dilution (Karlsen et al., 2022). For this experiment, VSEARCH (Rognes et al., 2016) in SEED2 was used to cluster ASVs into OTUs of 99% similarity. These OTUs were classified using GreenGenes2 supplemented with 16S rRNA genes from the salmon isolate genome data. These steps were necessary to improve occurrence across paired samples, reflect the inherent 16S rRNA gene variance within a given species, and increase species classification accuracy for the salmon-derived Vibrionaceae. The differential abundance analysis was performed in multiple ways utilizing DADA2 data (Nearing et al., 2022; Yerke et al., 2024). This included relative abundances, Hellinger-transformed relative abundances, and lognormal transformation as basic forms of transformation. In addition, CLR transformation was applied with the pseudocount set at 1 (Nearing et al., 2022) to account for compositionality. Limma-VOOM and DeSeq2 (Liu et al., 2021) were also used for analysis. Given that the samples are strictly paired, significance was determined using the Wilcoxon ranked sign test and paired t-tests. The false discovery rate (FDR) was controlled to manage false-positive occurrences (Benjamini and Hochberg, 1995). Effect size was determined by calculating Cohen's d and by calculating *r* (*r* = z/√N), where *z* is derived from the Wilcoxon signed rank test and *N* is the number of samples for a given feature (Fritz et al., 2012). The output significances and effect sizes were compared to determine consistency in features and also in relation to statistical power. Power was assessed in terms of sample number and effect size by using the SR_POWER function in the Real Statistics Package (v. 9.4.5.—C. Zaiontz). ASVs that showed significantly consistent abundance changes (based on the different transformations used) between gut sections had large effect sizes (*r* ≥ 0.7) to achieve a power of ≥0.7 (for a sample size of 27). In this respect, only OTUs with a paired sample size of ≥7 underwent analysis. OTUs with significant differences in occurrence in gut samples were then analyzed in a heatmap using Morpheus (Broad Institute).

### Colonization and virulence-based features annotated from genome data

Genes associated with host colonization phenotypes, including adherence, biofilm formation, quorum sensing, interbacterial competition, and host-directed virulence were surveyed in *Aliivibrio* as well as *Vibrio scophthalmi* genomes, supported by the automated Pannzer2 annotation. The survey of genes was enabled by mechanistic studies of gene and protein function performed to date, mostly in *A. fischeri* and *V. cholerae* strains, especially pertaining to colonization phenotypes (flagella, pili, adhesins, proteins associated with biofilm formation, quorum sensing, mucinase-like enzymes, relevant carbohydrate degradative pathways, and other functions). The gene surveys were conducted using the online databases KEGG (Kanehisa and Goto, 2000) and the VFDB resource (Liu et al., 2022). The presence of target proteins for this survey was assessed using protein comparisons with BLAST-P in NCBI, with confirmation for sequences based on similarity (>40%), length (>90%), and conserved domain structure.

## Results

### Multiyear surveys of gut microbial profiles

**Figure 1:**
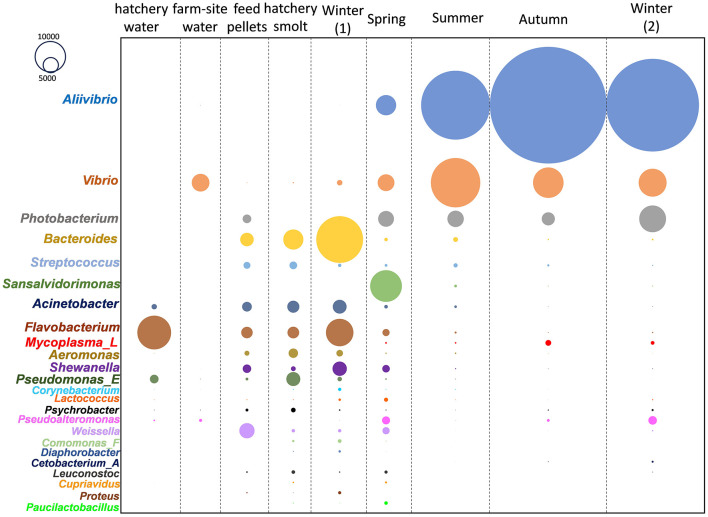
Changes in abundance (CLR) of predominant Atlantic salmon gut microbiome members in different seasonal periods after being transferred from freshwater hatchery to marine farms in southeast Tasmania.

The sampling covered fish reared from smolt to adults across six locations within the D'Entrecasteaux Channel region of Tasmania (Table 1, Supplementary Figure S1), with sampling occurring over annual seasons beginning in winter. This combined dataset was used to accurately determine which bacterial taxa consistently predominate each year and the shape of these responses. The collective data was subsampled and arrayed in terms of read abundances for the most abundant genera (Figure 1) and families (Supplementary Figure S2). *Aliivibrio, Vibrio*, and *Photobacterium* predominated in the salmon gut samples, while other bacterial genera were found in the hatchery smolt, feed, and water (Figure 1). ASVs classified as *Mycoplasma_L* (family Mycoplasmoidaceae) were the only other taxon to show any significant increase in abundance post-transfer and were not detected in the farm-site water. Based on relative abundance, *Aliivibrio* read numbers peaked in autumn samples, making up 60% of total reads compared to *Mycoplasma_L* at 3%. Collectively, *Aliivibrio, Vibrio, Photobacterium*, and *Mycoplasma_L* constituted 78% of total reads (of 4.0 × 10^7^ total reads from salmon digesta) and were also highly prevalent in samples (91–97% overall).

### Proliferating taxa in the salmon based on survey data

**Figure 2:**
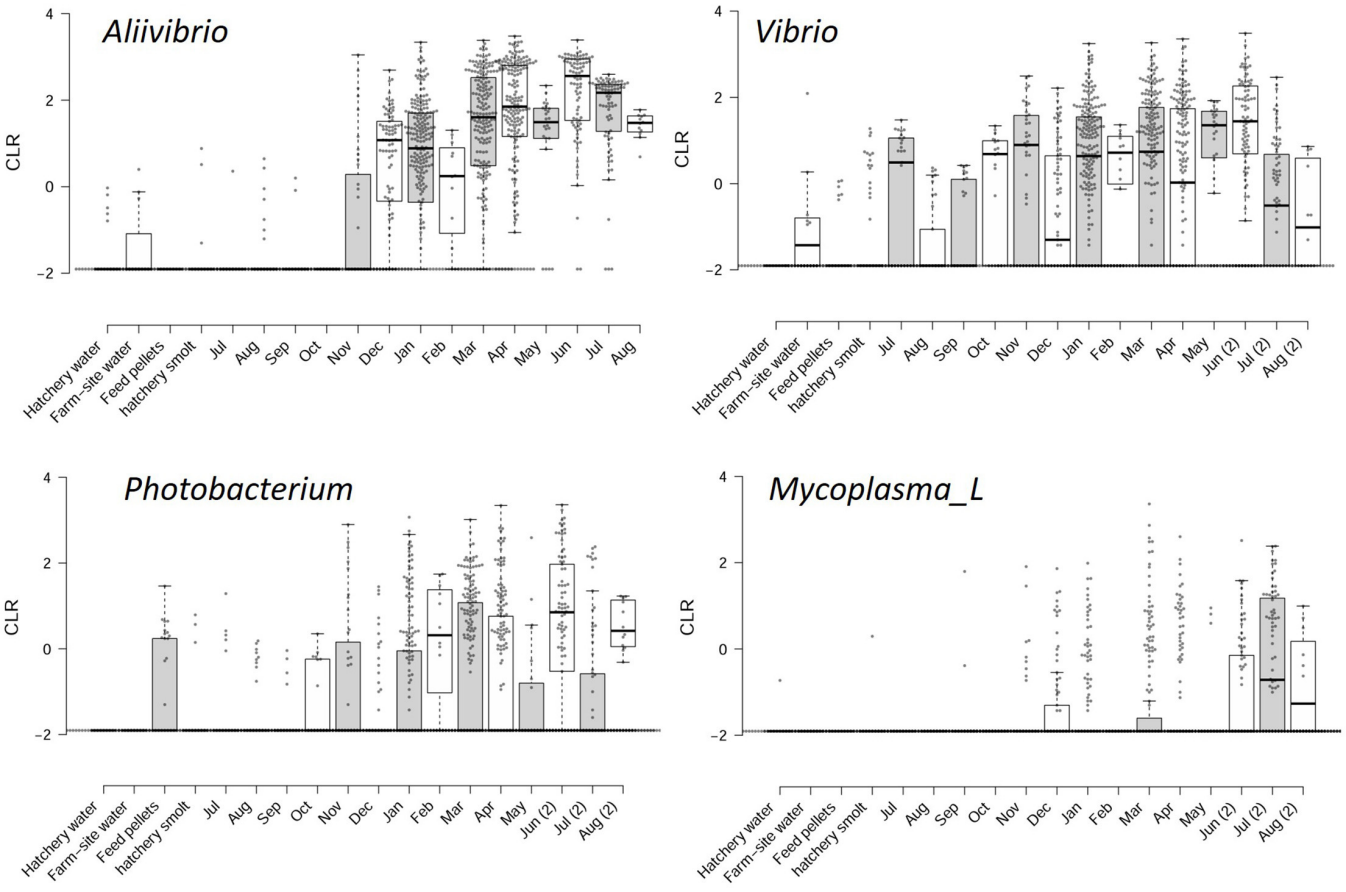
Sample CLR values for the most predominant taxa associated with the Atlantic salmon gut microbiome. The CLR values are compared to feed, water, and hatchery smolt, as well as monthly periods following the transfer from freshwater hatchery to marine farms in southeast Tasmania.

The abundance of these genera is shown in relation to the months fish spent at sea, amalgamating data from the surveys (Figure 2). Abundances were converted to CLR values to encapsulate relative abundance and show the variation between sampled fish. There is a high degree of variability; however, a clear pattern of increasing CLR abundance over time emerges. To define this difference more clearly, approximations of the differences in abundance were determined for samples collected just prior to marine transfer and during the first winter at sea, compared to samples taken after a 12-month period during the second winter at sea. By subtracting the mean CLR values between these samples, an approximation of population change on a log scale was obtained for all genera detected in the study (Supplementary Table S1). From these estimations, abundance increases by 3.8 (±1.5), 2.5 (±2.0), 1.8 (±1.6), and 2.6 (±2.2) log units for *Aliivibrio, Photobacterium, Vibrio*, and *Mycoplasma_L*, respectively (Supplementary Table S1). The marine genera *Propionigenium, Pseudoalteromonas*, and *Sansalvadorimonas* also increased in relative abundance (Supplementary Table S1), but these taxa were present at low abundance and their occurrence was less consistent between surveys. Based on this data, starting from a low abundance at the point of smolt input in the first winter, Vibrionaceae peaks after 6–8 months in the late summer-early autumn period, after which populations plateau. *Aliivibrio* exhibits the most definable growth-like response, with average CLR values reaching a maximal level by March to April (8 months after transfer; Figure 2).

### Cultivation-based observations and matching with ASV data

**Figure 3:**
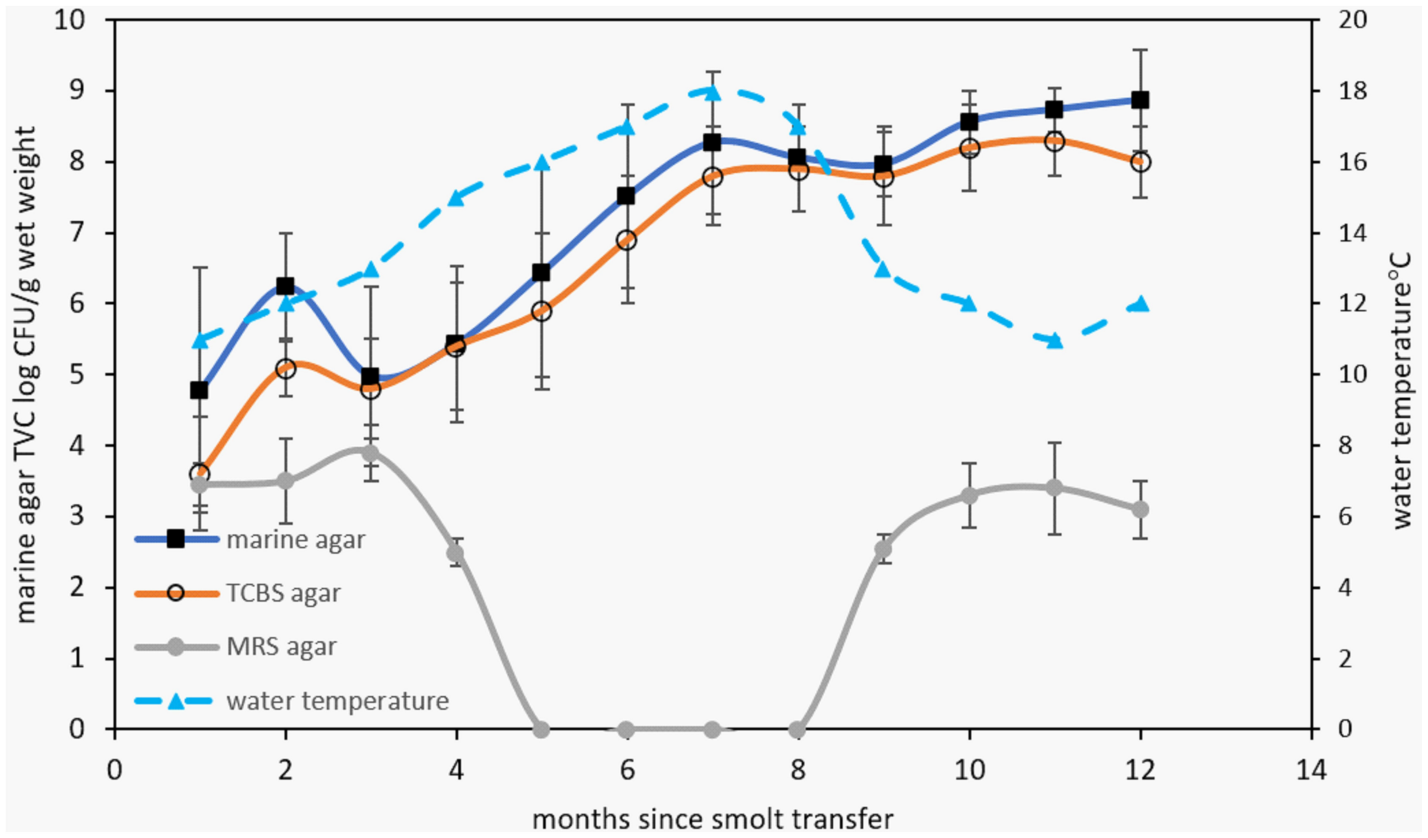
The top graph shows TVC data for marine, TCBS, and MRS agar (at 25°C) for bacterial populations obtained from Atlantic salmon digesta collected at separate times after smolt transfer to marine farms. Growth rates are shown relative to water temperature. MRS counts were below detection limits during the summer months. The bottom graph shows the square root of the specific growth rate of salmon gut isolates at different temperatures. The black squares and white circles represent growth in marine broth and marine broth containing 1% (w/v) bile salts.

The survey data responses were also compared with bacterial TVC data, measured from the smolt transfer period until the following winter for three of the survey years (Figure 3). The MA TVC data reached a maximum level of 8–9 log colony-forming units (CFU)/g sample wet weight within 7 months after smolt transfer (Figure 3). The close similarity observed between MA and TCBS count data (differing on average by 0.4 ± 0.4 log units) indicates that a high proportion of the TVC comes from bile salt-tolerant marine bacteria. Growth on MRS agar was found to be negative during the summer months but reached 3–4 log CFU/g in the cooler seasons. Based on distinct colony morphologies, isolates were then obtained from the MA and TCBS agar plates and purified for further analysis. Although the MRS colonies were not identified, Gram staining of the biomass from colonies suggested these could be lactic acid bacteria, being Gram-positive rods or coccibacilli that form pinpoint to 2 mm diameter white colonies.

A total of 96 salmon isolates were identified as *Aliivibrio* (68 isolates), *Photobacterium* (18 isolates), *Vibrio* (2 isolates), *Enterovibrio* (2 isolates), *Shewanella* (8 isolates), and *Psychrobacter* (1 isolate). The 16S rRNA gene sequence-based phylogenetic tree for these isolates is shown in Supplementary Figure S3. Most *Aliivibrio* isolates are grouped with *A. finisterrensis*, and two unclassified clusters are distinct from other *Aliivibrio* type strains (Supplementary Figure S3). *Photobacterium* isolates are grouped with the species *P. piscicola* and *P. toruni*, while the *Vibrio* isolates were most closely related to *V. scophthalmi*. The remaining isolates were most closely related to *Enterovibrio norvegicus, Shewanella pneumatophori*, and *Psychrobacter namhaiensis* (Supplementary Figure S3).

**Figure 4:**
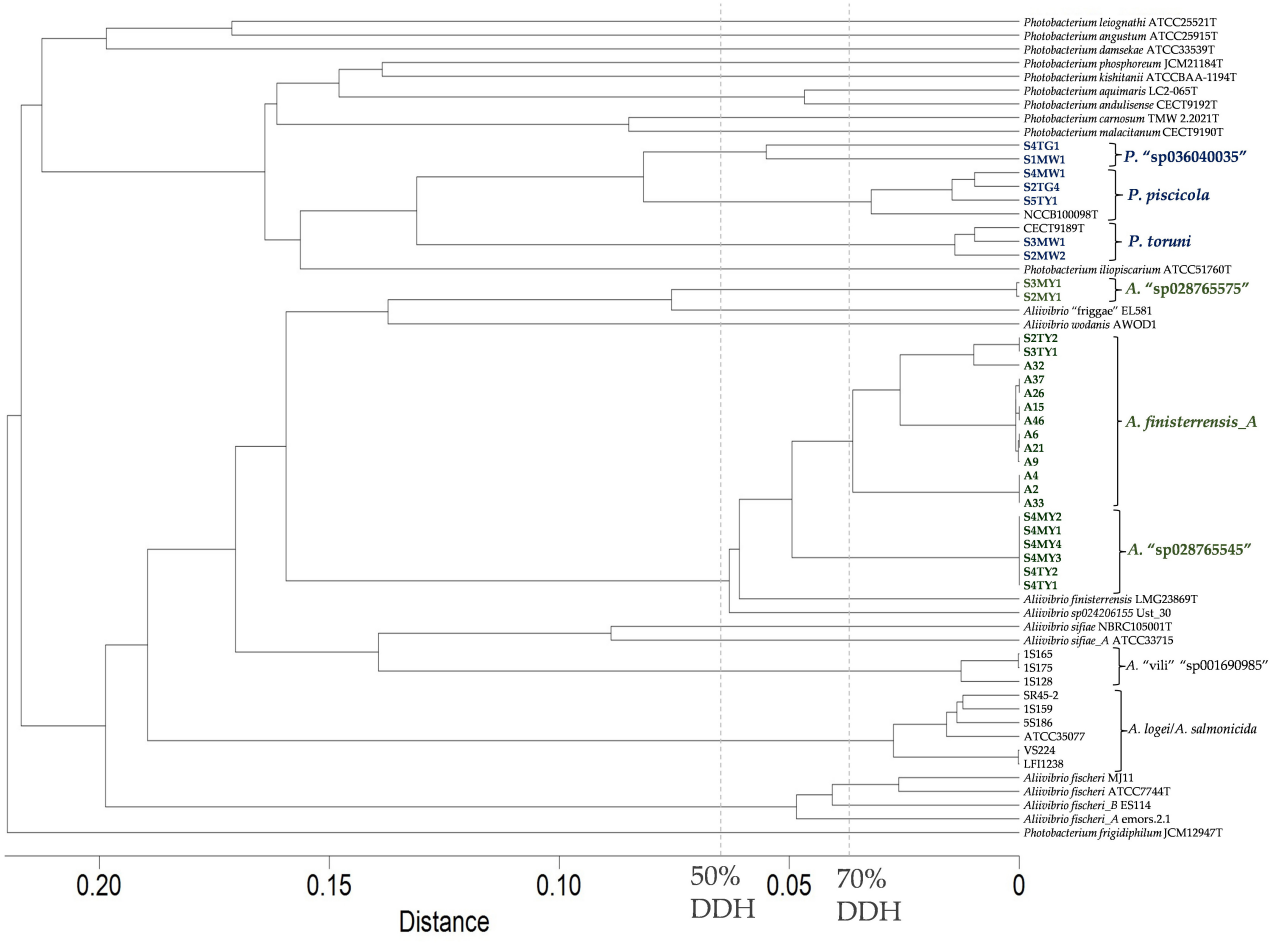
*In silico* DNA:DNA hybridization between *Aliivibrio* salmon isolates and other *Aliivibrio* strains using distances determined using GGDC model 2. The cladogram was created using complete linkage in Primer 7. A distance of 0.05 equates to an *in silico* DNA:DNA hybridization (DDH) similarity of ~60%.

Twenty-one of the *Aliivibrio* isolates and the type strain of *A. finisterrensis* (LMG23869T) underwent genome sequencing. Genome information is shown in Supplementary Table S2. Besides LMG23869T, the strains comprised five genetic groups designated as the A2, A9, A32, S2MY1, and S4MY1 strain groups (Figure 4). In the GTDB, these species are designated as *A*. “finisterrensis_A,” *A*. “sp028765545,” and *A*. “sp028765565.” *In silico* DNA: DNA hybridization (DDH) data, along with ANI data (Supplementary Figure S7), indicated that the A2, A9, and A32 groups represent a single species (DDH >70%, Figure 4; ANI >96%, Supplementary Figure S7). These strain groups are most closely related to the type strain of *Aliivibrio finisterrensis* (LMG23689T) based on ANI (95%) and DDH (58–65%). The S4MY1 group is equidistant from the A2/A9/A32 group cluster and the *A. finisterrensis* type strain (94% ANI, DDH 50–55%), while the S2MY1 group is a more distinct *Aliivibrio* species with ANI only at 80–87% (DDH 24–34%) with other available *Aliivibrio* genomes (Figure 4).

Seven *Photobacterium* salmon isolates were genome sequenced of which three strains belonged to *Photobacterium piscicola* and another two strains were members of *Photobacterium toruni* (Supplementary Table S2 summarizes the genome metric data). Strains S1MW1 and S4TG1 (DDH 58%, ANI 94.7%, Figure 4) represented unclassified species (GTDB—*P*. “sp036040035”). These species were most closely related to *P. piscicola* sharing an ANI of 92–95% and DDH 46–58%.

**Figure 5:**
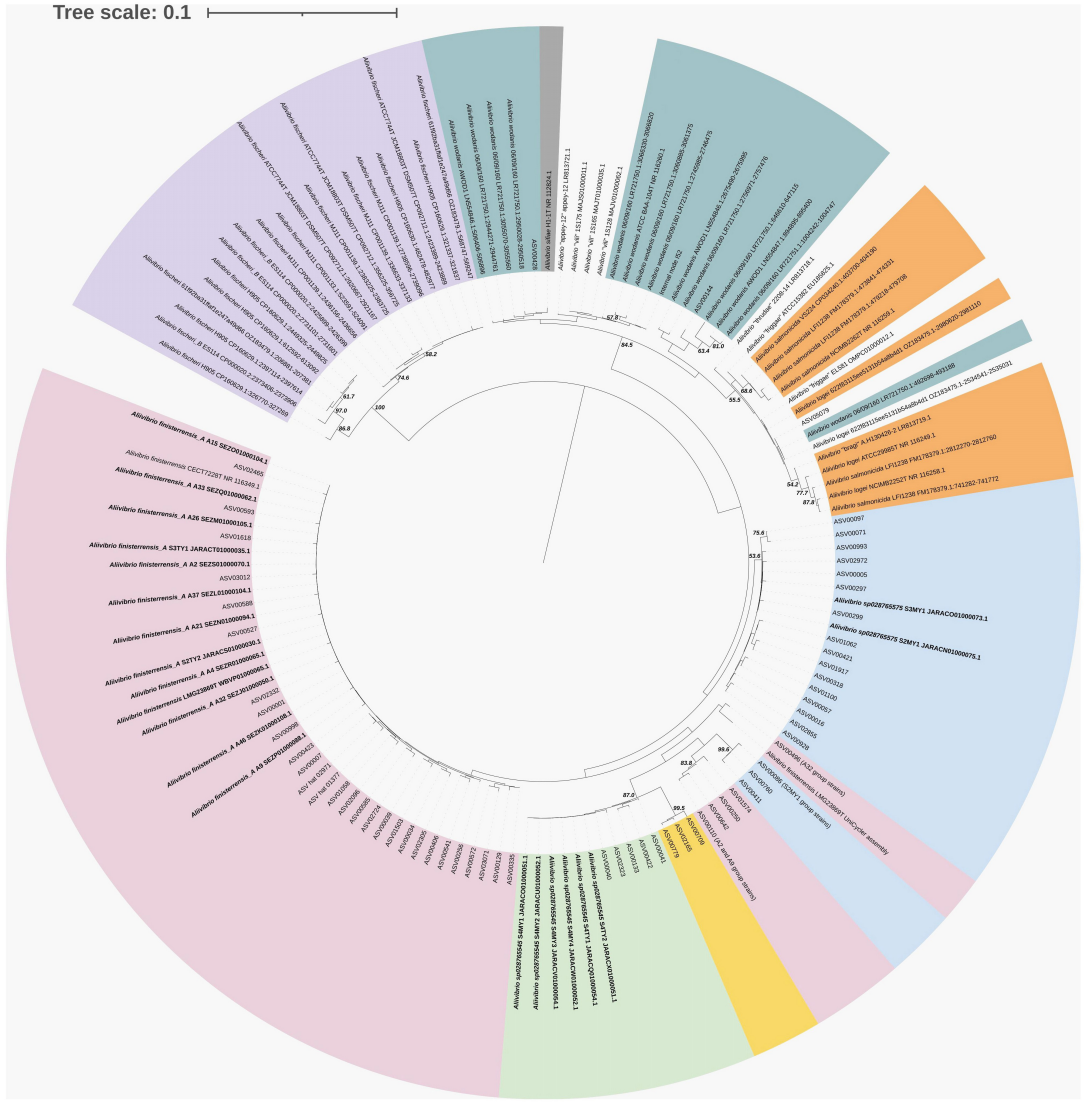
16S rRNA phylogenetic tree showing the relationship of *Aliivibrio* ASVs and isolates from surveyed farm fish in relation to sequences from strain genomes. Where no genome is available, the 16S rRNA gene from representative or type strains are included. The tree was created using maximum likelihood (Jukes–Cantor model) and BioNJ algorithms, and bootstraps were determined from 1,000 repetitions.

Comparisons were made between ASVs from the surveys and the isolate genome sequences for *Aliivibrio* (Figure 5), *Vibrio* (Supplementary Figure S4), and *Photobacterium* (Supplementary Figure S5). In the case of *Aliivibrio*, ASVs related to *Aliivibrio finisterrensis* and two related unclassified species represented the vast majority of *Aliivibrio* reads. Certain ASVs included in Figure 4 (shaded yellow) were determined to be variants from the genomes of representative *Aliivibrio* isolates. The details for these variants are shown in Supplementary Figure S6. The most abundant *Vibrio* ASVs were related to the species *Vibrio scophthalmi* and its close relatives; however, other *Vibrio* species are also present (Supplementary Figure S4). *Photobacterium* ASVs included those related to *P. piscicola* and *P. toruni* but also included sequences from at least one unknown species distinct from other *Photobacterium* type strains (Supplementary Figure S5). *Mycoplasma_L* reads were only represented by only a single ASV, with the closest relative being *Malacoplasma* species (89–90% similar).

### Distribution of mucosa and digesta associated bacteria within the GI tract of farmed Atlantic salmon

Raw sequence data was utilized from the study of Reid et al. (2024) for an analysis of the distribution of bacteria throughout the Atlantic salmon digestive tract, as described in detail in the methods section. Due to the summer and winter fish cohorts being sampled from separate farms, these were considered independent datasets. The fish were 26 weeks old (summer dataset) and 52 weeks old (winter dataset) post-transfer from hatcheries, with similar weights (2.9 kg) and condition factors (see Reid et al., 2024 for additional details). The OTUs (ASVs clustered at 99% similarity) included in the heat map were abundant and prevalent in the overall datasets, being present in at least 7 of the 27 fish sampled in the given comparisons. Multiple approaches were used to assess the significance of abundance differences, and the different approaches showed strong congruence (summarized in Supplementary Table S3), even if they were based on quite different transformation methods, i.e., proportional (Yerke et al., 2024), compositional (CLR), or a transformation intended for RNA sequence analysis (limma-VOOM, deseq2). After adjustment for multiplicity, it was observed that paired *t*-tests identified similar significant OTUs compared to those found using the Wilcoxon signed-rank test. Given the interest in this study regarding proliferating taxa in the gut of Atlantic salmon, focus is placed on the OTUs generating large positive effect sizes, as shown by the pale to bright red colors (Cohen's *d* > 0.5) in the heat map. Where species are mentioned in the heatmap (using GTDB taxonomy and placeholder designations), the sequence matches with Greengenes 2 were usually perfect (100%); if not, only the genus designation is provided.

**Figure 6:**
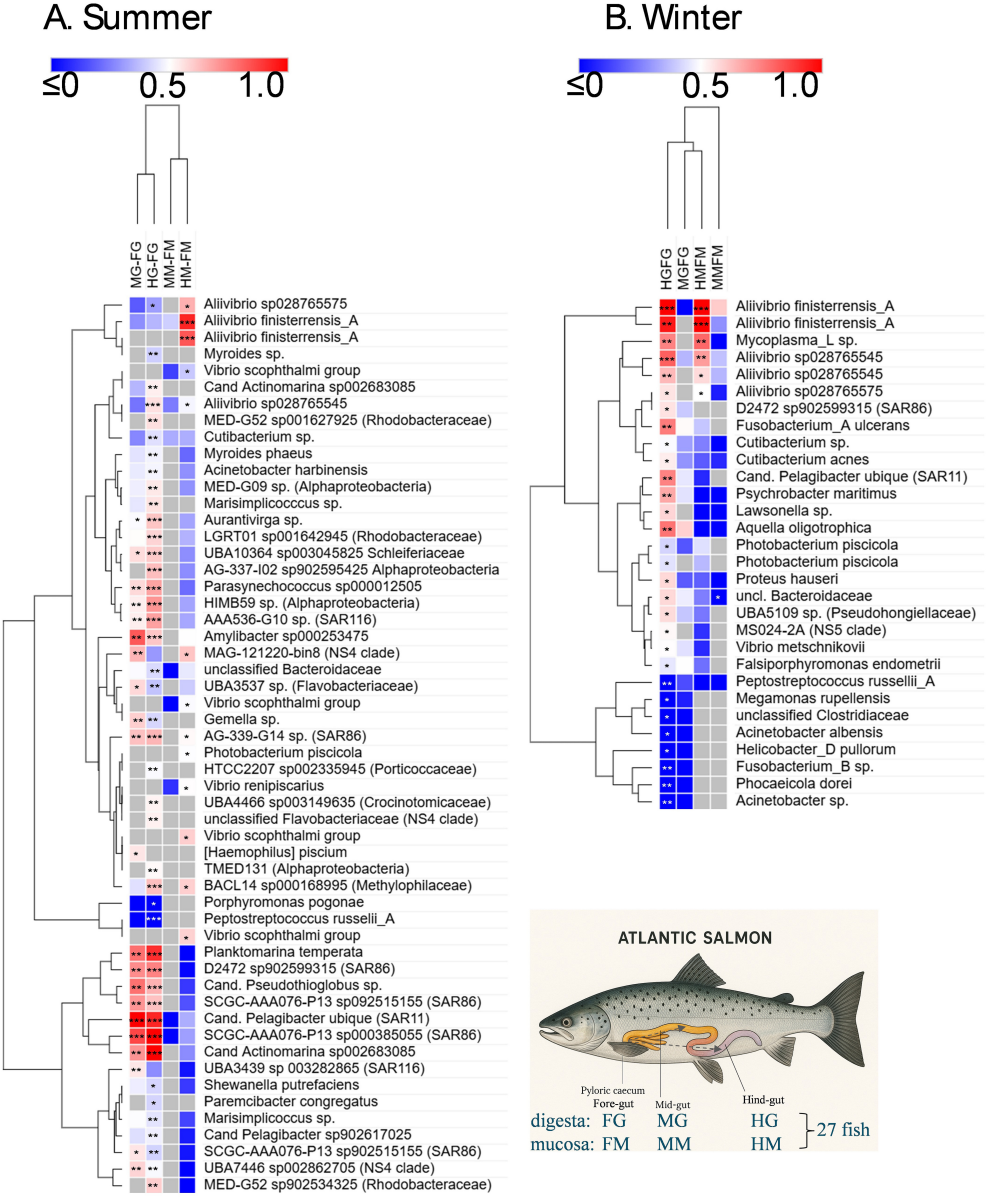
Heat map showing differential abundance of bacterial taxa (ASVs clustered into OTUs of 99% similarity) detected in the digesta (FG, foregut; MG, midgut; HG, hindgut) and mucosa (FM, foregut; MM, midgut; HM, hindgut) of different gut sections (FG, foregut; MG, midgut; HG, hindgut) of Atlantic salmon. Fish were collected in the summer and winter of 2018. The sampling immediately followed feeding and was done over a 24 h period (Reid et al., 2024) for 27 fish in each dataset. As indicated in the lower right schematic, digesta and mucosa of the mid- and hindgut were compared to equivalent samples from the foregut. The differences in abundances in these comparisons are indicated in the heatmap by the effect size (Cohen's *d*), with the color scheme centered on a value of 0.5. A red color indicates an increased abundance in the mid- or hindgut samples relative to the foregut samples. A blue color indicates only a slight increase in abundance through to a decrease in abundance. FDR-adjusted significant differences are indicated with asterisks: ***p < 0.01 for both the Wilcoxon signed-rank test and paired *t*-test; ** < 0.05 for both indicated tests; * < 0.05 for only one test. The OTUs were hierarchically clustered using Euclidean distances with complete linkage clustering.

For summer samples, abundances increased between the mid- and hindgut relative to the foregut in two groups of OTUs. One group showed strong increases (*d* = 0.70–1.25) for both MG-FG and HG-FG comparisons and included OTUs belonging to “*Candidatus* Pelagibacter” (SAR11 clade), “*Candidatus* Pseudothioglobus” (Gammaproteobacteria), “*Candidatus* Actinomarina” (Actinomycetota), D2472, and SCGC-AAA076-P13 (both in the SAR86 clade), as well as *Planktomarina* (family Rhodobacteraceae). The second group of OTUs only showed strong responses for HG-FG comparisons and included taxa from a range of marine genera (*Aurantivirga, Amylibacter, Parasynechococcus*, LGTR01, UBA10364, HIMB59, AG-337-IO, AAA536-G10). None of the aforementioned taxa were differentially abundant in the mucosal sample comparisons and were indeed orders of magnitude less abundant in mucosal samples compared to digesta samples. Instead *Aliivibrio finisterrensis*-related OTUs exhibited significant increases between HM and FM samples (*d* = 0.93 and 0.83). In the case of winter samples, taxa were found to show differentially abundant increases for hindgut digesta and mucosa relative to the foregut (HG-FG, HM-FM; Figure 6). These OTUs included *Aliivibrio finisterrensis*-related OTUs, *Mycoplasma*_*L*, and *A*. “sp028765545” (S4MY1 group strains; *d* = 0.69–1.42). A weaker response (*d* = 0.50–0.55) was observed for the other *Aliivibrio* salmon isolate group (S4MY1 group, *A*. “sp028765575”). *Vibrio* and *Photobacterium* OTUs exhibited differential abundance increases, but these were much weaker than those mentioned above. Multiple *Vibrio scophthalmi*-related OTUs exhibited increases in summer for mucosa (HM-FM, *d* = 0.42–0.60), while *Photobacterium piscicola* OTUs showed this response in both seasons (*d* = 0.41–0.51).

### Salmon-associated *Aliivibrio* growth attributes

Owing to the predominance of *Aliivibrio* in the gut samples and their association with the mucosal samples, an analysis of the basic growth and phenotypic properties (Table 2) was performed. The salmon isolates all have a mesophilic growth range (8–30°C), with an optimum growth temperature of 25–26°C. In marine broth, specific growth rates become slow (< 0.6 d^−1^) below 10°C (Supplementary Figure S8). All of the isolates require salt, which explains their absence in hatchery smolt and hatchery water (with a salinity of 0–2 psu). Optimal growth occurs at 20–25 psu (based on dissolved sea salts), with no growth occurring below 5 psu. The strains are bile salt tolerant (1–3% w/v ox bile salts) and can form biofilms on glass and polystyrene to varying degrees. Only the S4MY1 and A2 group strains were able to form significant biofilms on polystyrene within 2–3 d (Table 2). The isolates did not produce bioluminescence, except for two strains of the A32 group.

**Table 2:** Phenotypic data for *Aliivibrio* genetic sub-groups isolated from farmed Atlantic salmon.

**Phenotype**	**A2 group**	**A9 group**	**A32 group^a^**	**S4MY1 group**	**S2MY1 group**
Growth temperature range (°C)^a^	8–30 (25)	8–30 (25)	< 8–30 (25)	4–30 (25)	4–30 (25)
Salinity range for growth (sea salts % w/v)^b^	1.0–5.0 (2.5)	1.0–5.0 (3.0)	1.0–5.0 (2.5–3.0)	1.0–5.0 (2.5)	1.0–5.0 (2.5)
pH range for growth^c^	5.0–9.0 (6.5–8.3)	5.0–9.0 (6.5–8.3)	5.0–9.0 (6.5–8.6)	5.0–9.0 (6.5–8.6)	5.0–9.0 (6.5–8.6)
Maximum toleration of bile salts (% w/v)^d^	2.5	2.0–2.5	3.0	1.0–2.0	2.0–2.5
Bioluminescence	–	–	Varies	–	–
Biofilm formation on polystyrene/glass^e^	++/++	–/++	Varies (– to ++)/++	+++/+ to ++	–/++
Chitinolytic, carbohydrate fermentation	+	+	+	+	+

a Detectable growth (A600 (≥0.2 A_600_) occurring within 48 h of incubation. The approximate optimal temperature for growth is shown in parentheses; doubling time ranged from 4.2 to 6.0 h at this temperature. Strains S2TY2 and S3TY1 showed slight growth at 4°C within 48 h, but A32 did not. See Supplementary Figure S8 for modeled growth data.

b No growth occurs in the presence of ≤ 0.5% or ≥7.0% (w/v) sea salts; optimal salinity for growth is shown in parentheses.

c Values in parentheses denote luxuriant growth occurs within the indicated pH range.

d Oxoid ox bile salts no. 3 LP0056−50% w/v cholate; 50% w/v deoxycholate. The value shown is the solution concentration that allows detectable growth (≥0.2 A_600_ nm).

e The degree of biofilm formation is based on the crystal violet assay absorbance (A_550_ nm) values for growth developed after 24 h of incubation at 25°C (+++≥0.6, ++0.3–0.6, +0.1–0.3, – < 0.1). Strain A32 did not form biofilms on polystyrene and is non-bioluminescent.

### Genome-wide analysis for colonization-associated traits and toxin genes

The results support that *Aliivibrio* strongly colonizes the gut of Atlantic salmon, especially the mucosa. To begin to understand this, the genome data annotations of the *Aliivibrio* salmon isolate were mined for genes that could be linked to colonization and virulence phenotypes. The analysis was aided by available research data on the function of genes in *Aliivibrio fischeri* (Chavez-Dozal et al., 2012; Aschtgen et al., 2019; Visick et al., 2021), *Aliivibrio salmonicida* (Skåne et al., 2022), and various *Vibrio* spp., in particular *V. cholerae* (Cho et al., 2021), all of which are effective at forming biofilms in hosts. The results (Table 3) indicate that the salmon isolates have a wide array of genes linked to colonization that are often common across the genus *Aliivibrio* as well as the more distantly related species *Vibrio scophthalmi* (Table 3). In this respect, all of the isolates possessed homologs for a range of adherence factors including lytic chitin monooxygenase (*gpbA*, LPMO10B), outer membrane colonization factors (*ompU, ilpA*, MAM7), chitin-regulated pili (*pilABCD*), and MSH-type pili (*msh* genes). Curli fiber genes (*csg* genes) and tight adherence pili (*tad* genes) are present in specific strain groups. The isolates also have homologs for putative mucinases (*stcE*/*tagA*/*hapA* or *afcD*/*sslE* families). Most strain groups have syp-type, VPS-type, and cellulose polysaccharide synthesis gene loci, as well as genes associated with polysaccharide secretion (*eps* gene cluster). The isolates possess genes known to be mechanistically required for biofilm formation (*lapV*/*lapI, lapBCDEDG*) in *A. fischeri*. The A2, A9, A32, and S4MY1 salmon isolate groups have homologs of biofilm-associated genes that occur in *V. cholerae* but are absent in most other available *Aliivibrio* genomes. This includes rugosity factors *rbmABD* and *rbmC/bap1* that help generate a thick, resilient biofilm. All of the isolates possess regulatory genes known to control polysaccharide and biofilm formation (*hapR*/*litR, vpsR, vpsT, hbtRC*). Furthermore, most strains also possess type VI secretion (T6SS) systems and usually have multiple associated effectors (e.g., *hcp* and *vgrG*). Interestingly, strain groups S2MY1 and S4MY1 have genes for the multi-subunit cytolethal distending toxin (*cdtABC*; Table 3), which are most similar (50–55% amino acid identity) to genes found in pathogenic *E. coli* and *Salmonella enterica* (Supplementary Figure S9). Known virulence determinants for Vibrionaceae were otherwise absent. Other *Aliivibrio* strains have a range of toxins that could be identified.

**Table 3:** Host colonization relevant protein content in *Aliivibrio* species and *Vibrio scophthalmi* based on genome annotations and protein similarity analysis.

**Proteins**	**Protein function**	**Broad function (host)**	***A. salmonicida***	***A. logei***	***A. sifiae***	***A. fischeri***	***A. finisterensis***	**A2 Group**	**A9 group**	**A32/S2TY2 group**	**S4MY1 group**	**S2MY1 group**	***A. wodanis***	***A*. “*vili*”**	***A*. “*figgae*”**	***V. scophthalmi***
		**No. of genomes:**	**2**	**4**	**2**	**72**	**1**	**3**	**6**	**3**	**6**	**2**	**1**	**3**	**1**	**5**
GpbA (LPMO10A)	GlcNAc-binding protein	Adherence	100	100	100	100	100	100	100	100	100	100	100	100	100	100
LPMO10B	Chitin monooxygenase		100	100	100	100	100	100	100	100	100	100	0	100	100	100
OmpU	Outer membrane protein U		100	100	100	100	100	100	100	100	100	100	100	100	100	100
IlpA (MetQ)	Immunogenic lipoprotein A		100	100	100	100	100	100	100	100	100	100	100	100	100	100
MAM7 (PqiB)	Multivalent adhesion factor		100	100	100	100	100	100	100	100	100	100	100	100	100	100
PilABCD	Chitin-regulated pilus		0	100	100	94.4	100	100	100	100	100	100	100	100	100	100
MSHA gene cluster	MSH-type pilus		100	100	100	100	100	100	100	100	100	100	100	100	100	100
CsgABDEFG	Curli fibers		100	100	100	100	0	100	100	67	100	0	0	100	100	0
TadABCDEFG (Flp)	Tight adherence pilus		100	100	100	100	0	0	0	100	100	0	0	0	0	80
Tcp gene cluster	Toxin co-regulated pilus		0	0	0	2.8	0	0	0	0	0	0	0	0	0	0
AfcA	Accessory colonization factor	Host colonization	100	75	100	100	100	100	100	100	100	100	0	100	100	100
AfcB			0	0	0	0	0	0	0	0	0	0	0	0	0	0
AfcC			0	0	0	5.5	0	0	0	0	0	0	0	0	0	0
AfcD/SslE			0	0	0	11	0	0	0	0	0	100	0	0	0	0
NanA operon	Neu5AC degradation		100	100	100	97.2	0	100	0	0	0	0	0	0	100	100
StcE/TagA/HapA	Mucinases (putative)		0	100	100	24.3	100	100	100	100	100	100	100	100	100	100
NanH	Sialidase		0	0	0	0	0	0	0	0	0	0	0	0	0	0
KatA	Class II peroxidase		100	100	100	100	100	100	100	100	100	100	100	100	100	100
NorRVW	Nitric oxide reductase		100	100	100	100	0	0	100	100	100	0	0	0	100	0
EroS	Glucosaminoglycan lyase	“Aphrodisiac” other?	0	0	50	100	0	100	0	0	0	0	0	0	100	0
LuxA	Lux system	Bioluminescence	100	100	100	100	100	0	0	67	0	0	0	0	100	0
LuxI homologs	AHL synthesis	Quorum sensing	100	100	100	84.7	100	0	0	67	100	0	0	0	100	0
AinS homologs	AHL synthesis		100	75	100	100	0	100	100	100	100	100	100	100	100	0
Eps/Gps gene cluster	T2SS EPS system	Biofilm formation	100	100	100	100	100	100	100	100	100	100	100	100	100	100
Syp gene cluster	Syp polysaccharide		100	100	100	100	0	100	100	100	100	100	100	100	100	100
BscABC	Bacterial cellulose synthesis		50	100	100	100	0	100	100	100	100	100	0	0	0	100
Vps gene cluster	Vps polysaccharide		0	0	0	0	0	100	100	100	100	0	0	0	0	0
RbmABCD, Bap1	Rugosity/biofilm structure		0	0	0	0	0	100	100	100	100	0	0	0	0	0
LapV/LapI	T1SS adhesins		100	100	100	100	0	100	100	100	100	100	0	100	100	100
LapBCEDG	Biofilm modulation system		100	100	100	100	100	100	100	100	100	100	100	100	100	100
BdlA	Biofilm dispersal		0	0	0	2.8	0	100	0	0	0	0	100	0	0	0
HapR/LitR	Biofilm repressor		100	100	100	100	100	100	100	100	100	100	100	100	100	100
VpsR	Biofilm positive regulator		100	100	100	100	100	100	100	100	100	100	100	100	100	100
VpsT	Biofilm positive regulator		100	100	100	100	0	100	100	0	100	0	0	100	100	100
HbtRC (TcpPH)	Habitat transition regulator		0	75	100	80.6	0	0	100	100	100	100	0	0	0	0
T6SS	T6SS VAS system/effectors	Cell competition	100	100	100	100	0	100	100	100	100	0	100	100	0	100
CNF-1	Cytotoxic necrotizing factor 1	Toxins/virulence	100	100	100	2.8	0	0	0	0	0	0	0	0	0	0
Hap/Vva	Vibriolysin		0	100	100	0	0	0	0	0	0	0	100	0	0	0
RtxABCD	RTX toxin		0	100	0	0	0	0	0	0	0	0	0	0	0	0
Tdh/Trh	Thermostable direct hemolysin		0	0	50	1.4	0	0	0	0	0	0	0	0	100	0
Tlh	Thermolabile hemolysin		0	0	0	0	0	0	0	0	0	0	100	0	0	0
CdtABC	Cytolethal distending toxin		0	0	0	0	0	0	0	0	100	50	0	0	0	0
Ace	Accessory cholera enterotoxin		0	0	0	2.8	0	0	0	0	0	0	0	0	0	0
Zot/Zot-like	Zonula occludens toxin/homologs		0	0	0	11	0	0	0	0	0	0	0	0	100	40
VCC/VvhA-like	Leukocidin family toxin		0	0	100	29.1	0	0	0	0	0	0	0	0	0	20

Colored values represent the percentage of strains containing the indicated protein homolog having >40% similarity and >80% of length to the reference protein. Of the 74 A. fischeri genomes, 3 represent the type strain (ATCC 7744 = DSM 507 = JCM 18803) and are considered as one strain for the analysis. AfcB and AfcD homologs are present in most strains but only have 35–40% similarity with homologs from V. cholerae. PilC homologs are absent in certain A. fischeri and A. salmonicida strains. The tad operon may be present in two separate alleles, but three alleles are rare. MSHA genes mshE, mshP, and/or mshQ may have diverged homologs in specific strains. The tcp gene cluster occurs in the A. fischeri ES114 and VCW2E2 genomes, but both lack tcpR. Two or three StcE/Tag-like homologs may be present in a given strain. GAG (glycosaminoglycan) lyases of the PL8 family are present in A. logei IS159 and all three “A. vili” strains (1S128, 1S165, 1S175), though these proteins share only 38–40% homology with the GAG lyase EroS of A. fischeri ES114. The Vps cluster gene vpsC is not present in any strain, while additional genes in the cluster are found in specific strains, e.g., strain A32. A. fischeri strains may have a Zot domain-containing protein, except ES114 and VCWE2E, which have adjacent Zot and Ace homologs.

## Discussion

Based on the analysis of the main taxa present in the Atlantic salmon GI tract digesta and mucosa, the predominant taxa are similar to those observed in Tasmanian Atlantic salmon, as indicated in a summary of relevant studies (Supplementary Table S4). From this broader perspective, the presence of Vibrionaceae is most evident when surface seawater (or seawater tank) temperatures exceed 12–13°C, particularly in smolt or post-smolt sampled weeks to months after deployment to a marine location (Godoy et al., 2015; Bozzi D Rasmussen et al., 2021; Lorgen-Ritchie et al., 2021; Brealey et al., 2022; Schaal et al., 2023; Dhanasiri et al., 2023; Huyben et al., 2020; Li et al., 2021). In the case of Tasmanian farmed salmon, we observed that *Aliivibrio, Vibrio, Photobacterium*, and *Mycoplasma_L* were the only taxa that consistently increased in abundance over time during the 9-year survey period, being detected in all of the surveys. The approximated increase of 2–3 log units (based on CLR data) and the MA/TCBS TVC data suggest that obvious abundance increases can be attributed to growth in the gut. The TVC data based on traditional agar support these findings by providing a population scale to work from. Consequently, Vibrionaceae clearly expand and predominate by late summer to early autumn, a process that takes about 6–8 months. After this period, overall populations decline slightly but remain stable during the winter months following the summer/autumn peak. Other taxa that demonstrated increases (*Propionigenium, Sansalvidorimonas, Pseudolateromonas*) were observed only in certain surveys; for example, *Propionigenium* was detected in the early surveys (2010–2011, 2011–2012), while *Sansalvidorimonas* was detected in the 2017–2018 surveys.

The species of the main proliferating taxa, especially of the genus *Aliivibrio*, were analyzed in detail. We obtained isolates of the predominant *Aliivibrio* species present in the salmon, as well as *Vibrio* and *Photobacterium*. These isolates were obtained from high dilutions of samples on MA and are thus highly abundant in the digesta samples (>7 log units/g for digesta from summer fish). Genome sequencing revealing 16S rRNA gene variants aided the identification of ASVs, including certain reads that were found to be variant 16S rRNA gene sequences (Supplementary Figure S6). The study by Vera-Ponce de León et al. (2024) identified *Aliivibrio* species that were predominant in a set of saltwater fish from a Norwegian coastal site; this included *Aliivibrio salmonicida, A. finisterrensis*, and two other *Aliivibrio* species. From the results here, Tasmanian salmon contain quite different predominant *Aliivibrio* species. Most strains are related to *A. finisterrensis* (A2, A9, A32, and S4MY1 groups), while a novel group (S2MY1 group) is most closely related but still distinct from the placeholder species *Aliivibrio* “friggae” (Klemetsen et al., 2021).

Based on the ANI and *in silico* hybridization data, the A2/A9/A32 group strains represent a single species that is closely related but distinct from *A. finisterrensis* if the species boundary guidelines are strictly interpreted (Bach et al., 2023). However, the 16S rRNA genes of the strains are indistinguishable from the *A. finisterrensis* type strain. The main *Vibrio* species present in Tasmanian farmed salmon were also different from those in Norwegian salmon (Vera-Ponce de León et al., 2024), with the species *V. scophthalmi* predominant instead of strains more closely related to *V. splendidus*. *Photobacterium* found in Norwegian salmon includes the species *P. phosphoreum*; however, in Tasmanian salmon, the predominant species were *P. piscicola* and *P. toruni*. *Shewanella* and *Psychrobacter* are also relatively abundant, as found in both the Vera-Ponce de León et al. study and this study. The species-level differences could relate to the sea surface temperature, the (epi)genetics of the salmon (Hansen et al., 2023), and an implicit amplification effect of strains becoming abundant over time within the D'Entrecasteaux Channel region due to the high concentration of active Atlantic salmon farms. The heightened abundance means they are more likely to colonize than less abundant species, as suggested previously (Heys et al., 2020).

We establish here that the proliferating taxa, in particular *Aliivibrio*, in the Atlantic salmon samples occur in abundance in washed mucosal samples and show an increasing abundance trend from the foregut to the hindgut. The data analyzed included summer and winter fish cohort specimens since there was an interest in investigating the effect of seasonal temperature differences on gut microbiome dynamics in more detail (Reid et al., 2024). In this respect, *Aliivibrio* salmon bacterial isolates from Tasmanian salmon only grow slowly below 10°C (< 0.6 d^−1^, Figure 5). This temperature is the minimum winter temperature recorded in the past decade in the main salmon-growing region of Tasmania (Supplementary Figure S1). Based on laboratory culture results, the growth rates of *Aliivibrio* in spring and summer, when water temperatures are >15°C, should exceed 1.0 d−1. In winter, water temperatures (~10–13°C in southeast Tasmania) result in populations that seem to be slightly lower than the summer peak due to slow growth. Since warmer seawater conditions would enhance the growth rates of Vibrionaceae, it is expected that more intense summer sea surface temperatures could promote the rapidity of the colonization process and result in greater biomass levels in fish. It is unknown how microbial diversity would be affected, for instance, by the appearance of only one predominant species. Potentially, stochasticity could be influential in the assembly of gut microbiomes in salmon. The colder waters typical for northern hemisphere salmon farming regions (i.e., Norwegian annual range is 11–17°C in summer and 4–5°C in winter) could explain the contrast in the species makeup of Norwegian and Tasmanian farmed salmon. Based on our data, if water temperatures drop below 8–10°C, *Aliivibrio finisterrensis* and S2MY1 group-related strains should become less abundant.

Examining the distribution of colonizing taxa in paired gut samples provided more targeted information on microbiome dynamics and structural relationships. Differences between mucosa and digesta were highlighted, as well as the effects of seasons. Importantly, mucosal washed samples are not affected by the feed background, as indicated by the lack of detection of chloroplast and mitochondria-derived 16S rRNA sequences originating from feed. On this basis, the data indicate that few taxa seem to actively colonize the mucosal layer in Atlantic salmon. Within the digesta samples from summer fish, a range of OTUs became clearly more abundant, showing high effect sizes for both the mid- and hindgut to foregut comparisons. These OTUs include seawater bacteria such as SAR11, SAR86, SAR116, NS4, and NS5 clades, Rhodobacteraceae, marine Actinomycetota (“*Candidatus* Actinomarina”), and Cyanobacteriota (*Parasynechococcus*). These taxa are typical seawater specialists, either strictly oligotrophic heterotrophs or phototrophic. They are usually unculturable on agar media and have streamlined genomes. To date, there is no evidence that such bacteria have direct functional relevance or impact in Atlantic salmon; their occurrence is purely due to the fish taking up seawater during feeding. There is typically a high water content in digesta samples (Zarkasi et al., 2016). Digesta water content was found to be greater in summer fish than in those examined during winter (Reid et al., 2024), which supports why seawater bacteria predominate in digesta in summer. There is a distinct possibility that the increased abundance of seawater bacteria is due to water concentration effects. The “drinking” rate is higher in salmon adapted to seawater and during active feeding, and it is used for the purposes of osmoregulation (Eddy, 2007), maintaining a level of salinity in the gut lumen. The effect of water uptake could lead to seawater bacteria having a greater overall representation in the microbiome data. This occurrence has also been suggested as a reason that seawater-dwelling Vibrionaceae are able to colonize the gut (Bjørgen et al., 2020), since the salinity is suitable for their growth, with most species being halophilic (Table 2). The indicated seawater specialists are common in Tasmanian waters, especially during summer (Brown et al., 2024).

Since most sea-going wild salmon and those that have returned to rivers also possess considerable levels of *Vibrionaceae* (Supplementary Table S4, Llewellyn et al., 2016), colonization by Vibrionaceae is clearly a natural process. The observation that *Aliivibrio* and *Mycoplasma_L* proliferate in the mucosa suggests these taxa have traits consistent with an avidity for salmon colonization. We have previously hypothesized that *Aliivibrio* colonizers are a cause of fecal cast production in fish sampled during summer (Reid et al., 2024). Fecal cast production in finfish has been attributed to disease or stress (Breen et al., 2021; Dhar et al., 2017; Birrell et al., 2003; Figueroa et al., 2017; Skår and Mortensen, 2007). The expected higher growth rates and biomass of *Aliivibrio* and *Vibrio* spp. in summer may increase the risk of dysbiosis or disease, but this depends on the species' capacity to negatively affect the host. At this time, the causal relationships remain unresolved. A preliminary attempt to understand these responses resulted in the observation of colonization- and virulence-relevant mechanisms in *Aliivibrio* genomes (Figure 3). A nearly universal trait of Vibrionaceae is motility, which is necessary for initial adherence in the gut as well as spreading throughout (Homma et al., 2022). The salmon-associated bacterial strains examined were all motile, and based on the genomic data, this is driven solely by polar flagella. Homologs of flagellin genes forming lateral flagella (*laf* genes found in *Vibrio vulnificus*), which could aid in spreading in viscous fluids, in or on the host (Homma et al., 2022), were not found in any *Aliivibrio* genome. Vibrionaceae also produce different types of pili and a range of other specific proteins to enable stable adherence to host gut cells and mucosa. Lytic chitin monooxygenase proteins LPM010A and LPM010B (Skåne et al., 2022; Wong et al., 2012), chitin-regulated pili (Paranjpye and Strom, 2005), and MSHA-type pili (Teschler et al., 2015), were found in all *Aliivibrio* strains and have been shown in *A. fischeri* to be mechanistic elements for adherence and establishing populations in squid host light organs (Visick et al., 2021). The salmon isolates also possessed genes for curli fiber synthesis (*csg* operon; Karan et al., 2021), while 1 or 2 alleles for the tight adherence pili (Tad operon; Pu and Rowe-Magnus, 2018) were present in specific strain groups (A32, S4MY1 groups). This type of pili may also enhance stable adhesion. Potentially, other proteins could also be involved in adherence, as found for *V. cholerae*, and are broadly conserved in Vibrionaceae, e.g., IlpA/MetQ, OmpU, and MAM7/PqiB (Lee et al., 2011; Goo et al., 2006; Yang et al., 2018; Krachler et al., 2011; Table 3). A homolog of the outer membrane accessory colonization factor gene *afcA*, which influences spreading in the host (Hughes et al., 1995; Cai et al., 2018), is universally present. Homologs of the other accessory colonization genes (*afcB, afcC, afcD*), putatively linked to mucin chemotaxis and degradation (Valiente et al., 2018), are not evident in most *Aliivibrio* strains; however, diverged homologous genes (35–40% identity) do occur. The salmon isolates possess putative mucinases, including YghJ/SslE/AfcD (Nesta et al., 2014; Szabady et al., 2011) and/or StcE/TagA (Rossiter and Wuest, 2017) family lipoprotein metallopeptidases. Adding to a probable capability of colonizing and degrading mucin and other proteins that make up the gut epithelial layer are putative chondroitinase-like enzymes (polysaccharide lyase family 8). These glycosaminoglycan degradative enzymes are co-located with genes coding for enzymes that degrade D-glucuronate, N-acetyl-D-glucosamine, and N-acetyl-D-galactosamine in certain *Aliivibrio* species. Chondroitinase activity (*eroS* gene) by *A. fischeri* has been shown to induce sexual reproduction in a choanoflagellate (Rossiter and Wuest, 2017; Woznica et al., 2017), but the relevance of this capability in gut colonization remains to be demonstrated. The *eroS* gene and associated metabolic pathway genes are strain group-specific and were found in the A2 strain group. The potential ability to utilize N-acetylneuraminate (*nanA* operon; Boyd et al., 2015) and other carbohydrates liberated from mucins (N-acetyl-D-glucosamine, D-galactose, L-fucose) is also present in certain *Aliivibrio* species. Among the salmon isolates, the *nanA* operon is found in the A2 group strains. All genomes surveyed have a *katA* homolog shown to be needed by *A. fischeri* for dealing with host-driven peroxide defenses (Visick and Ruby, 1998) and also possess nitric oxide reductase (NorRVW), which could protect cells from host NO synthesis (Gardette et al., 2020).

Biofilm formation and compatibility with the host are key for colonizing bacteria to establish stable populations in fish. Bacteria not native to the host, such as commercial probiotic strains, may only colonize transiently and less efficiently due to their inability to form a stable biofilm (Li et al., 2018). As found in *A. fischeri*, the salmon isolates and other *Aliivibrio* species have similar genes for biofilm formation (Christensen et al., 2020; Fung et al., 2024). This includes a type I secretion system (T1SS), which delivers the adhesin LapV and proteins of the Lap adhesin delivery system (operon *lapBCEDG*; Christensen et al., 2020) that are used to establish active biofilms *in vivo*. In addition, the salmon isolates possess a conserved T2SS for exopolysaccharide (EPS) delivery. Salmon isolates have genes for the syp polysaccharide, the main EPS formed by *A. fischeri* to create mature host biofilms (Dial et al., 2021). The gene locus of the syp polysaccharide was found to be syntenic (in relation to gene loci in *A. fischeri* strains) in the salmon strains and most other *Aliivibrio* species. Similarly, conserved regulators for biofilm matrix formation are present, such as the quorum sensing/competence repressor HapR/LitR and VspR (Bjelland et al., 2012; Cohen et al., 2021). Some genes were noted to be specific to species or sub-species strain groups (Table 3). These traits contribute to colonization and may influence host specificity. For example, cellulose synthesis genes (*bsc* operon, Abidi et al., 2022) are present in the genomes of 75% of *Aliivibrio* genetic groups, while homologs for the VPS polysaccharide locus of *V. cholerae* (Schwechheimer et al., 2020) were found only in the genomes of the A2/A9/A32 and S4MY1 group strains. The VPS polysaccharide contributes to a viscous polysaccharide that enables *V. cholerae* and other *Vibrio* to colonize a range of ecosystems and hosts (Fung et al., 2024). Specific genes in this locus differ in the salmon isolates compared to *V. cholerae*, and the synteny is not fully preserved, suggesting that the polysaccharide formed could have different chemical properties. Furthermore, the same strains possess adjacent genes that may regulate biofilm thickness and structure, as found for *V. cholerae* (*rbmABD, rbmC*/*bap1*; Fong and Yildiz, 2007). Quorum sensing systems in the salmon isolates are controlled by the synthesis of different acylated homoserine lactones (AHLs) and autoinducer-2 (LuxS system; Lupp and Ruby, 2004). In this respect, all of the salmon strains possess *luxS* and have genes for two AinS, LasI, or LuxI family AHL synthetases (Table 3). This suggests that the salmon isolates (and most other *Aliivibrio*) may be able to form an array of quorum sensing-related chemical messengers, as is the case for *A. fischeri* (Girard et al., 2019).

Virulence gene distribution was of considerable interest, particularly in discovering genes that might be linked to fecal cast production (Reid et al., 2024). The survey examined genes flagged generically as virulence factors as well as T6SS systems that have been suggested to be involved in host virulence, not only in interbacterial competition (Rubio et al., 2019). In this respect, all of the salmon strains possess a T6SS system, except for the S2MY1 group, and have multiple associated effectors (Crisan and Hammer, 2020; Cohen et al., 2023). Strains A9 and A21 possess both *hcp* and S-type pyocin-like (Scholl, 2017) homologs on a contig that could be a large plasmid. It is currently unknown if T6SS has any direct effect on hosts; however, the T6SS in *A. fischeri* is used effectively to become predominant in squid hosts (Guckes and Miyashiro, 2023). In comparison, toxin genes are sporadically distributed across *Aliivibrio*. For example, a minority of *A. fischeri* strains possess genes for CNF-1, Tdh, Ace, VCC/VvhA-like leucocidin, and/or Zot/Zot-like proteins found in various pathogenic *Vibrio* (Cai et al., 2018). Surprisingly, *cdtABC* genes coding the subunits for CTD (Lai et al., 2021) occur in one S2MY1 group strain (S2MY1, but not S3MY1) and in all six of the S4MY1 group strains. The nucleotide *and* amino acid sequences for the three Cdt subunits were unexpectedly found to be identical between the isolates and distinct from homologs of *E. coli* and *Salmonella* (~50–55% amino acid homology), as well as from homologs found in a single *V. cholerae* isolate 633012 (Supplementary Figure S9). The genes occur in a region of chromosome II where extensive transposase/integrase gene decay has occurred. The most populous salmon strain groups (A2, A9, and A32) completely lack any known genes linked to a cytotoxic function.

The overall results indicate *Aliivibrio* possesses various colonization-relevant genes that are group and species-specific. This suggests that colonizer genetic groups could have different colonization specificities and preferences. Various features found in *Aliivibrio* are also shared by *Vibrio scophthalmi* (Table 3). This species is a successful colonizer of fish and is sometimes an opportunistic pathogen (Zhang et al., 2020). The differences in gene distribution may affect adherence, mucin/mucosal interactions, signal transduction, and biofilm structure within different hosts, thus determining specificity, success rates, and persistence of colonization. At this stage, the genomic data offers only a surface view; the details of the colonization process still need to be resolved. The data provided in this study suggest a series of proteins and phenotypes that could be further researched in this respect. For example, putative mucinases detected in the salmon colonizers are particularly interesting. The *Vibrio cholerae* HapA mucinase facilitates penetration and detachment into the mucosal layer and may also release substrate sugars and amino acids (Silva et al., 2006). Few of the Atlantic salmon isolates seem to be able to degrade neuraminate, a major glycocomponent of intestinal mucins of Atlantic salmon (Jin et al., 2015). Mucinase activity by colonizer strains could primarily allow persistence in the mucosal layer (Silva et al., 2006). However, the combined mucinase secretion activity of the colonizer community could liberate a range of glycopeptides, amino acids, and sugar residues that can be used as nutrients by the microbiome as a whole. In this respect, all colonizer strains could grow on chitin and possess chitinolytic enzymes (Table 2; LPMO10A, LPMO10B, Table 3), which have been shown to be important in mucosal layer adherence and required for virulence (Skåne et al., 2022; Wong et al., 2012). Gut mucins of Atlantic salmon typically contain N-acetyl-D-glucosamine (Benktander et al., 2019), and chitinase-like enzymes can remove these residues from mucin (Skåne et al., 2022). Salmon mucin is also rich in galactose residues, and based on the genome annotations, salmon isolates were found to possess the Leloir galactose metabolism pathway. Furthermore, salmon isolate groups also synthesize multiple types of polysaccharides that may be involved in the colonization of marine animals and abiotic surfaces. It is possible that these polysaccharides co-contribute to biofilms during the colonization of fish. *Aliivibrio* genetic diversity may also impact other biological aspects of colonization, such as quorum sensing regulation. The types of AHL synthetases differed between the colonizer genetic groups and can even differ within the same group; for example, the A32 group strains have either LuxI or AinS family AHL synthetases. In terms of the gut ecosystem, *Aliivibrio* strains may be able to respond to AHLs of other strains, allowing rapid responses in gene regulation while simultaneously maximizing fitness and competitiveness during colonization and growth. Potentially linked to signal transduction are pathogen-like functions by which colonists could exploit host cells. The occurrence of virulence genes among *Aliivibrio* species, however, is quite sporadic and less conserved than other traits examined (Table 3). The presence of CDT-gene positive strains in salmon isolates is intriguing but raises questions. Are the genes expressed *in vivo*? Do they have a functional role that does not involve harming the host—for example, aiding persistence in the salmon gut—or are they merely unstable genes derived from recent horizontal gene transfer events? The distribution of CDT is presently unknown in marine bacteria but is extremely rare. The fact that the genes are identical between two different *Aliivibrio* genetic groups suggests that lateral gene transfer involved the same source and occurred during a similar period. The genomic region around the cdtABC operon, was found to be rich with pseudogenes and remnant transposase genes. This suggests that the transfer of genes could have occurred within a mobile element that is now decayed. Further research is clearly needed to link fish and bacterial biology in controlled experiments to better understand how *Aliivibrio* and other colonizing taxa interact with Atlantic salmon. This study suggests pathways for such ventures, including adherence and biofilm formation, AHL-based signal transduction, mucosal interactions (mucinase production), competition between gut microbiota, potential impacts on host cells by T6SS effectors, and finally potential toxin production by specific strains (e.g., CDT toxin).

## Conclusions

We found specific Vibrionaceae occurring in high abundance in the GI tracts of Atlantic salmon farmed in Tasmania. Although the same genera are present, we found that the species differed from those seen in northern hemisphere farmed salmon. The gut profile survey revealed a restricted range of species, mostly novel, that are robust colonizers of the mucosal layer. Such strains could be a focus for future studies related to Atlantic salmon gut function and health, especially concerning climate change. This is due to the colonization patterns of *Aliivibrio* seemingly being driven by water temperature. The most common *Aliivibrio* colonists lacked any obvious virulence factors, which is good news; however, other abundant species were found to have specific virulence genes, including notably genes for the CDT toxin. Understanding the significance of such genes in relation to Atlantic salmon health and productivity, as well as the biology of gut colonization, is a logical next step. Combining temporal sampling with the genomic biology of cultivated colonists proved particularly useful in defining the dynamics of colonizing bacteria and identifying traits that may be relevant to salmon health and welfare. The findings revealed interesting and complex traits among different *Aliivibrio* spp. genetic groups that contribute to their success in colonizing salmon.

## Data Availability

The raw 16S rRNA gene sequence data and metadata files are deposited at the NCMI SRA database under the Bio Project codes listed in Table 1. *Aliivibrio* and *Photobacterium* genome sequence data are deposited in the NCBI database under WGS project codes WBVP00000000 (*A. finsterrensis* LMG 23689), SEZJ00000000 to SEZQ00000000 (*Aliivibrio* strains A32, A46, A37, A26, A21, A15, A9, A33, A4, A2), JARACN00000000 to JARACX00000000 (*Aliivibrio* strains S2MY1, S3MY1, S4MY1, S4TY1, A6, S2TY2, S3TY1, S4MY2, S4MY3, S4MY4, S4TY2), and JAYXUB00000000 to JAYXUK00000000 (*Photobacterium piscicola* strains S1MW1, S2TG4, S4MW1, S5TY1; *P. toruni* strains S2MW2, S3MW1; *Photobacterium* sp. S4TG1). Additional information is shared at Mendeley Data at https://data.mendeley.com/drafts/pymm9hg9s3).

## Supplementary material

Supplementary Figure S1 Location of farms from which Atlantic salmon were sampled.

Supplementary Figure S2 Proportion of bacterial taxonomic groups at the family rank across the survey data including water, feed, and gut samples.

Supplementary Figure S3 Identification of Atlantic salmon bacterial gut isolates based on 16S rRNA gene sequence comparisons to type strains. The tree was created using maximum likelihood (Jukes–Cantor model) and clustered using neighbor-joining (BioNJ).

Supplementary Figure S4 16S rRNA phylogenetic tree showing the relationship of *Vibrio* ASVs in relation to sequences from strain genomes. Where no genome is available, the 16S rRNA gene from type strains is included. The tree was created using maximum likelihood (Jukes–Cantor model) and BioNJ algorithms and visualized with ITOL.

Supplementary Figure S5 16S rRNA phylogenetic tree showing the relationship of *Photobacterium* ASVs in relation to sequences from strain genomes. Where no genome is available, the 16S rRNA gene from type strains is included. The tree was created using maximum likelihood (Jukes–Cantor model) and BioNJ algorithms and visualized with ITOL.

Supplementary Figure S6 Aligned 16S rRNA variants within the V1–V3 region for available *Aliivibrio* species, including those from this study. Variants were detected from raw reads using ASV data as a guide. This was used to identify *Aliivibrio* related ASVs from DADA2 that grouped separately from the species cluster.

Supplementary Figure S7 Average nucleotide identity (ANI)-based cladogram showing the taxonomic relationship of sequenced salmon isolates. The cladogram was created using complete linkage in Primer 7. The dashed line denotes the notional species cut-off of 95%.

Supplementary Figure S8 Growth rates over the temperature range for *Aliivibrio* salmon isolates.

Supplementary Figure S9 Comparison of concatenated cytolethal toxin subunit CdtA, CdtB, and CdtC protein sequences from Atlantic salmon *Aliivibrio* isolates with Gram-negative pathogens possessing Cdt.

Supplementary Table S1 Changes in CLR abundance over time.

Supplementary Table S2 Genome Information.

Supplementary Table S3 Cross-sectional differential abundance data.

Supplementary Table S4 Summary of studies examining the gut microbiome of Atlantic salmon.
