# Supplementary material for: Characterization of bacteria colonizing the mucosal layer of the gastrointestinal tract of Atlantic salmon farmed in a warm water region
Source: Front Microbiol. 2025 Jul 23;16:1564052. doi: 10.3389/fmicb.2025.1564052 (PMC12327903; doi:10.3389/fmicb.2025.1564052)
Supplement: Supplementary Figure S1 — Location of farms from which Atlantic salmon were sampled. [file Data_Sheet_1.pdf]

Characterization of bacteria colonizing the mucosal layer of the gastrointestinal tract of Atlantic salmon farmed in a warm water region

Supplementary Figures S1 to S8.

Chantelle E Reid[1], Josephine Hamlett[1], Qi Zhi Tan[1], Richard S Taylor[2], Andrew Bissett[2], Barbara F Nowak[3], John P Bowman[1]*

[1]Tasmanian Institute of Agriculture, University of Tasmania, Hobart, Tasmania 7005, Australia

[2]Commonwealth Scientific and Industrial Research Organisation, Hobart, Tasmania 7005, Australia

[3]Institute of Marine and Antarctic Studies, University of Tasmania, Hobart, Tasmania 7001, Australia

*Corresponding author

John P. Bowman. Centre for Food Safety and Innovation, Tasmanian Institute of Agriculture, University of Tasmania, Hobart, Tasmania 7005, Australia. Email: john.bowman@utas.edu.au, Telephone: +61 03 6226 6380.

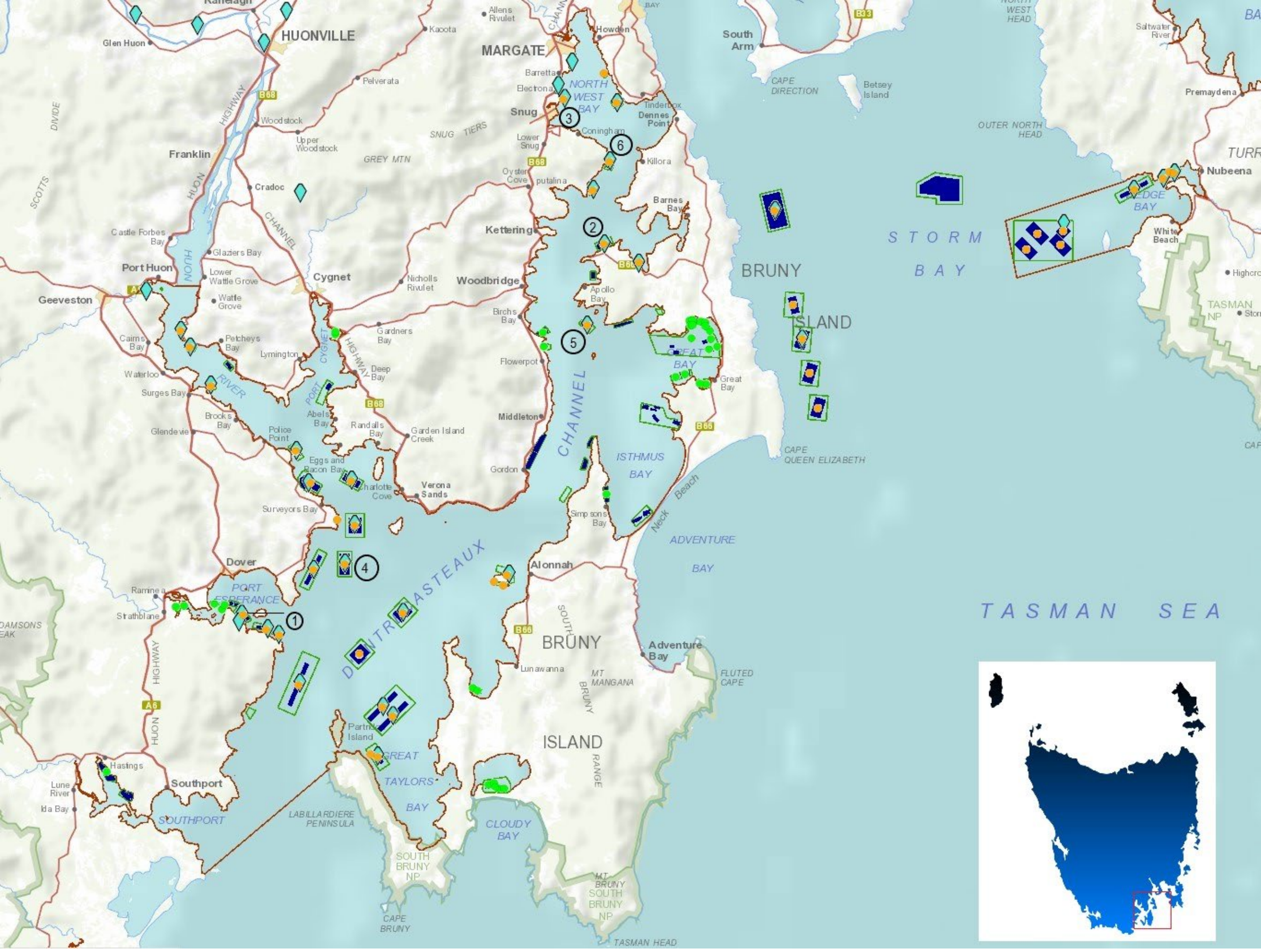

Figure S1. Location of farms from which samples were collected for this study and antecedent published studies as shown in Table 1. Farms sampled were at (1) Meads Creek, Port Esperance; (2) Robert's Point lease, D'Entrecasteaux Channel, (3) North West Bay; (4) Red Cliffs, D'Entrecasteaux Channel; (5) Soldier's Point, D'Entrecasteaux Channel; Sheppard's lease, D'Entrecasteaux Channel (6).

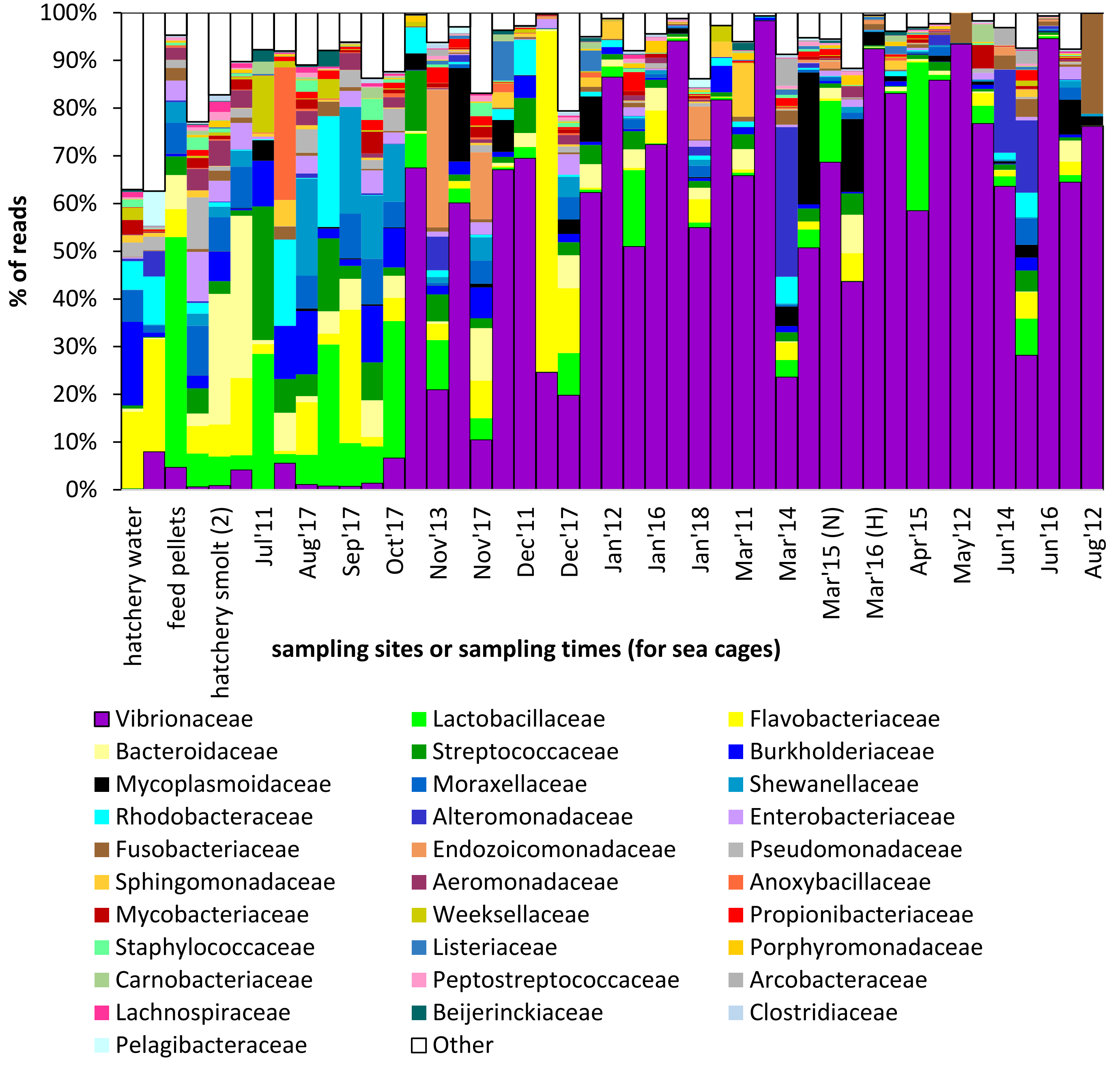

Figure S2. The proportional representation of bacterial taxa from farmed Atlantic salmon fish gut samples collected over several surveys in Tasmania (2010 to 2018, Table 1) compared to hatchery and farm-site water, feed pellets and hatchery smolt digesta. More details are also shown in Supplementary datafile 1. For the 0-3 to 10-13 months all profiles shown derive from fecal squeeze samples.

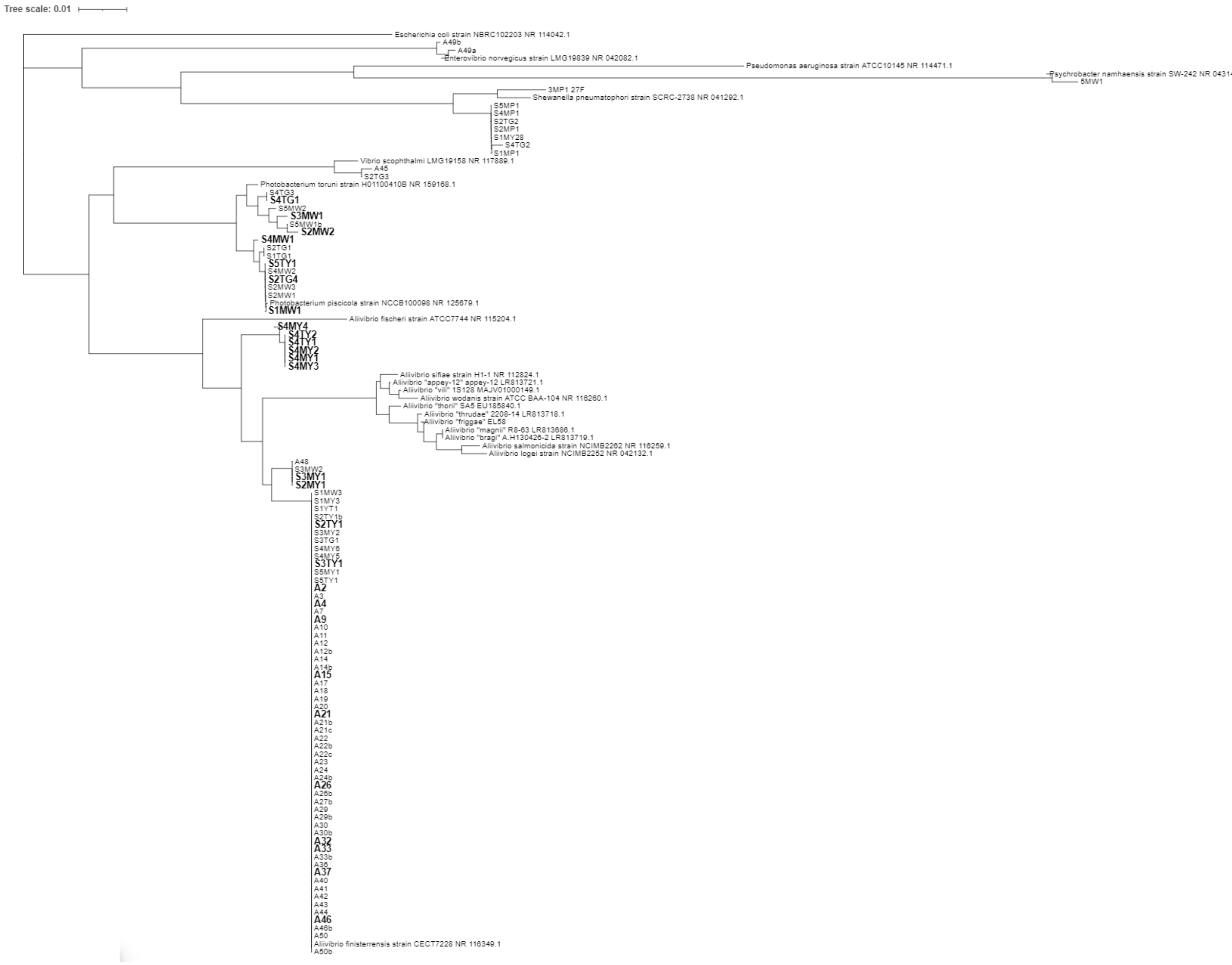

Figure S3. 16S rRNA based phylogenetic tree showing the closest related validly described species to bacterial strains isolated from farmed Atlantic salmon from south east Tasmania. Strain designations shown in bold type were subsequently selected for genome sequencing. The tree was created using phylogeny.fr using PhyML (JC model) and BioNJ.

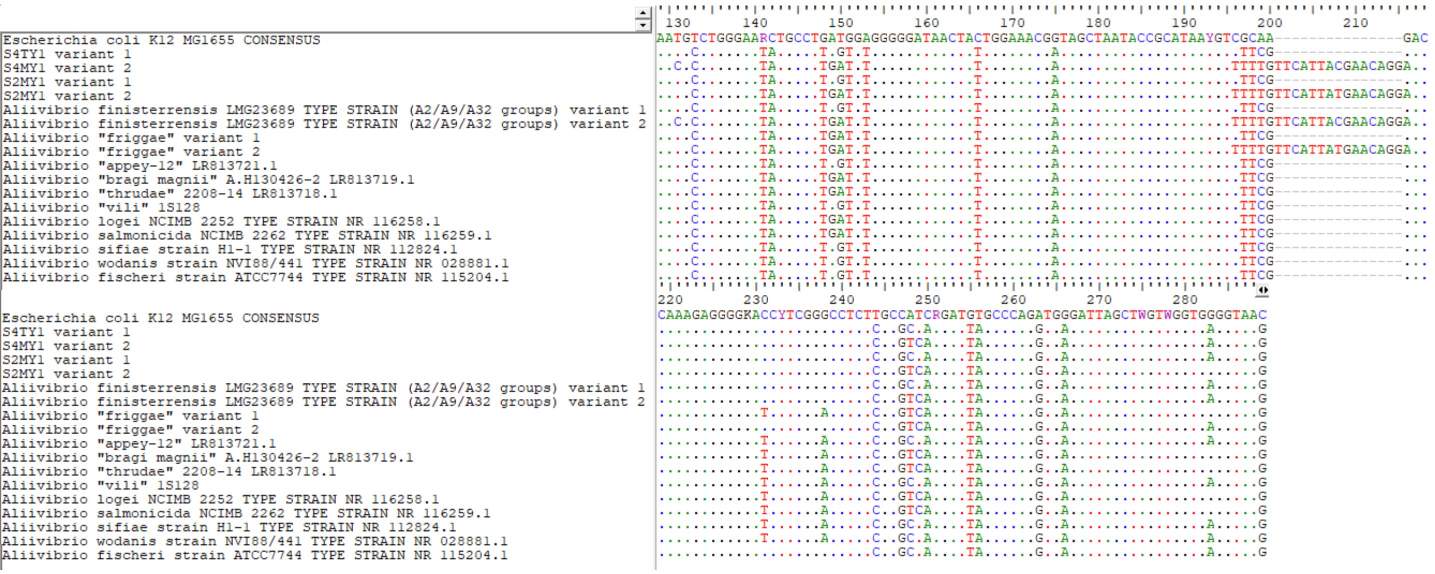

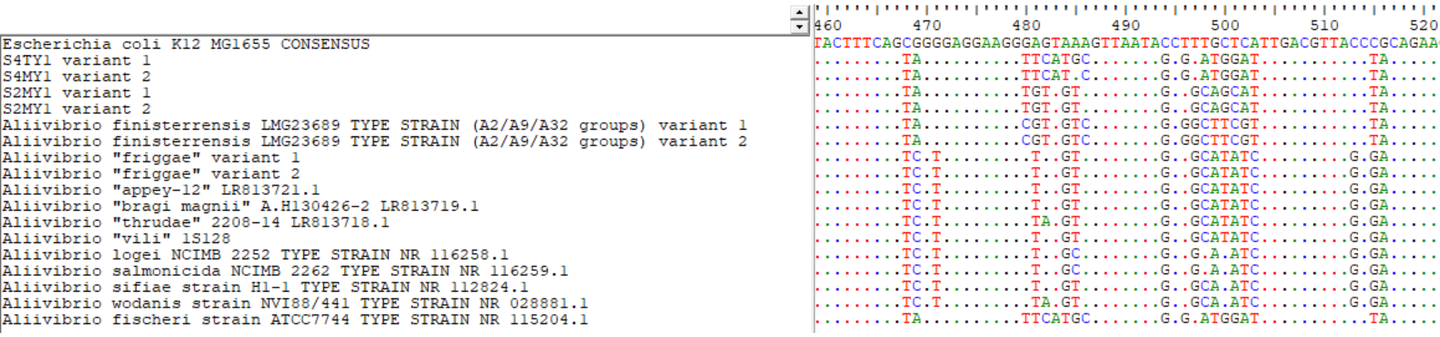

Figure S4. Alignment of sections of the V1-V3 region of 16S rRNA genes (*E. coli* numbering equivalent 10 to 534) for validly described *Aliivibrio* species and placeholder designations. All sequences are derived from genomes or from cultured isolates via direct PCR amplification. The two main variants are shown for 3 major Atlantic salmon gut isolate groups (*A. finisterrensis*, S2MY1, S4TY1) as well as showing regions of hypervariability in the V1-V3 region. Variant sequences were added to the Silva 138.1 database used for classifying reads in the farm survey aspect of the study. this enabled better identification of species within the limits of resolution.

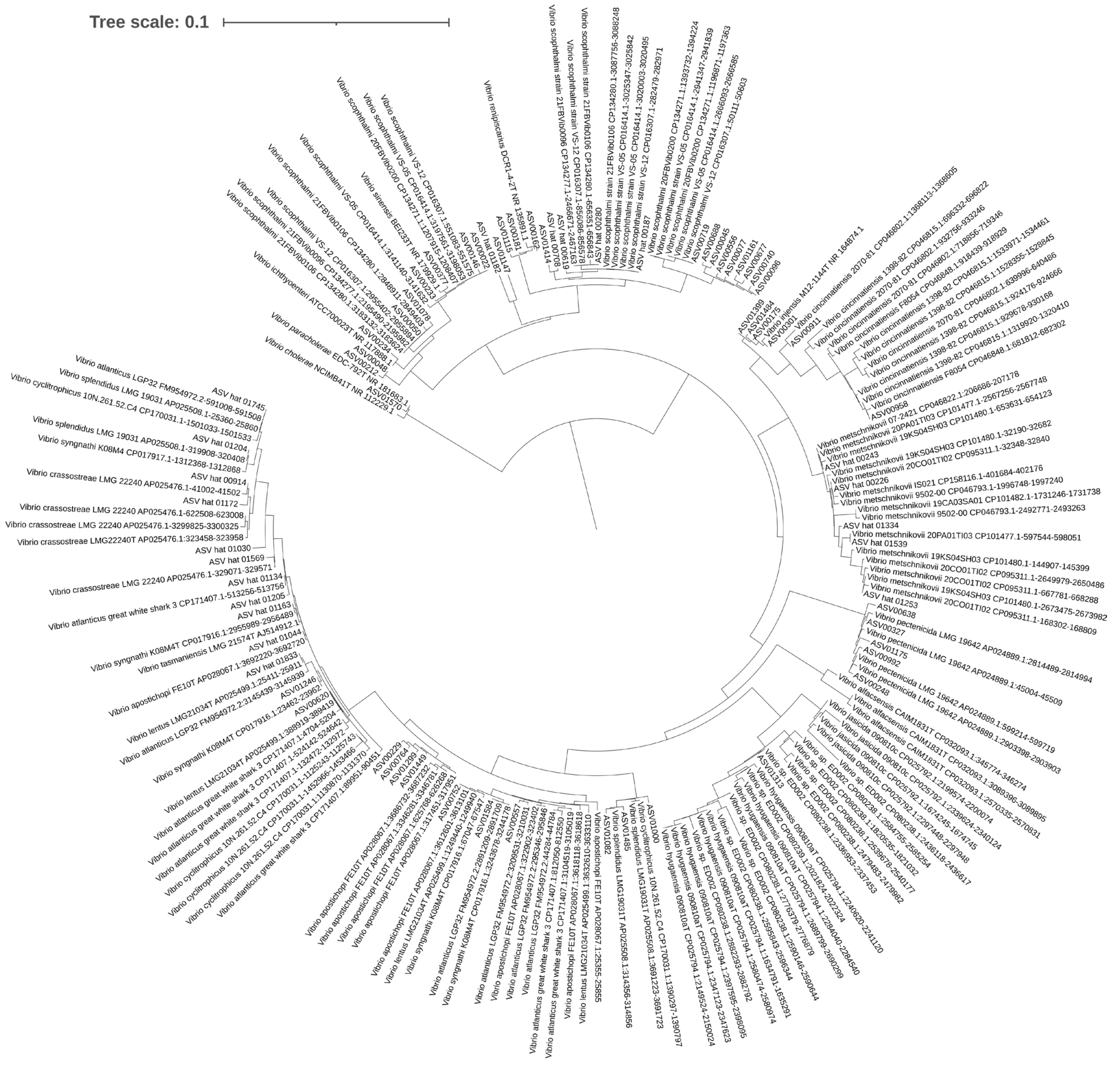

Figure S5. 16S rRNA based phylogenetic tree showing the closest related validly described *Vibrio* species to ASVs obtained from farmed Atlantic salmon from south east Tasmania. The tree was created using phylogeny.fr using PhyML (JC model) and BioNJ.

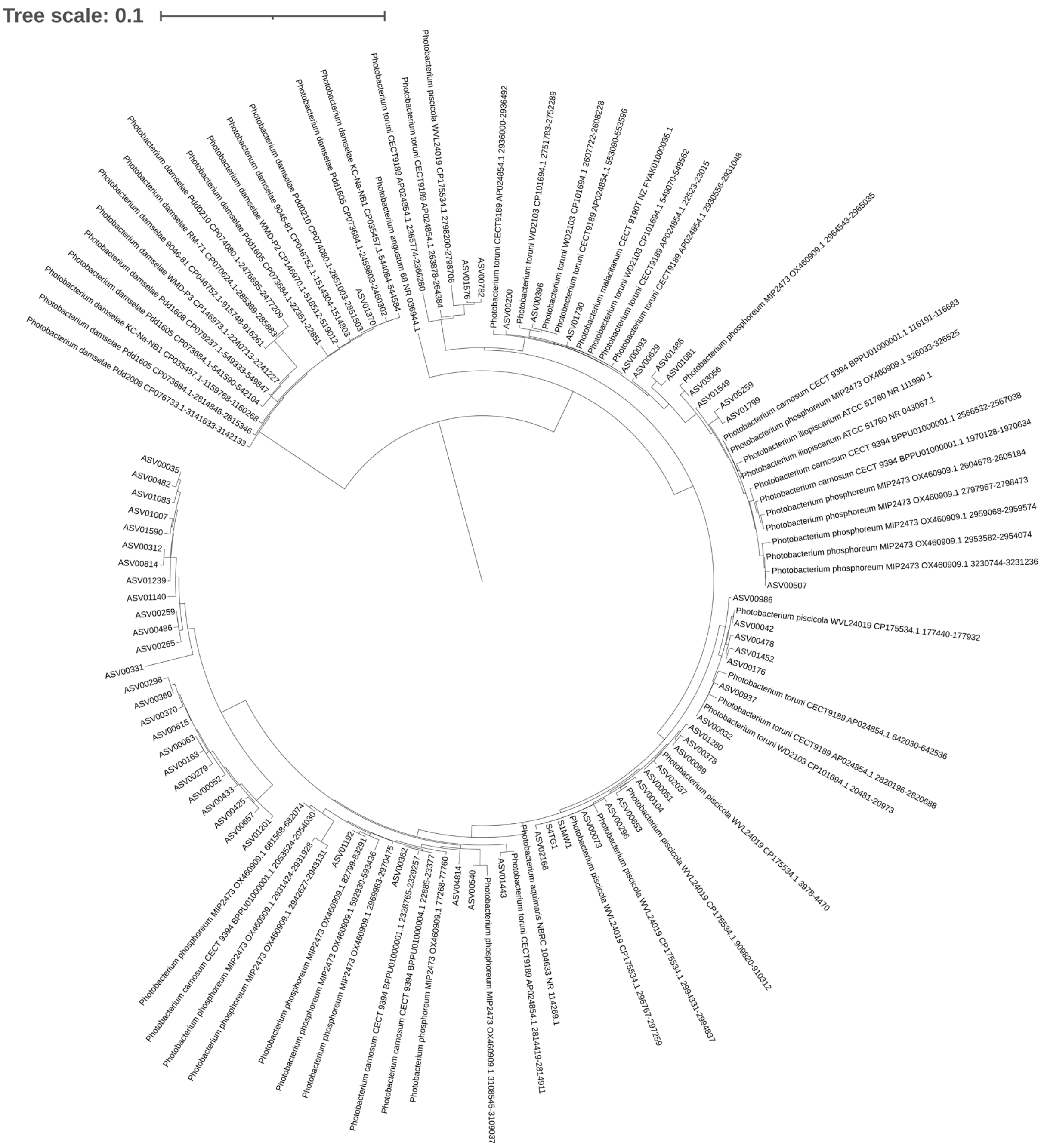

Figure S6. 16S rRNA based phylogenetic tree showing the closest related validly described *Photobacterium* species to ASVs and isolated strains (S4TG1, S1MW1) obtained from farmed Atlantic salmon from south east Tasmania. The tree was created using phylogeny.fr using PhyML (JC model) and BioNJ.

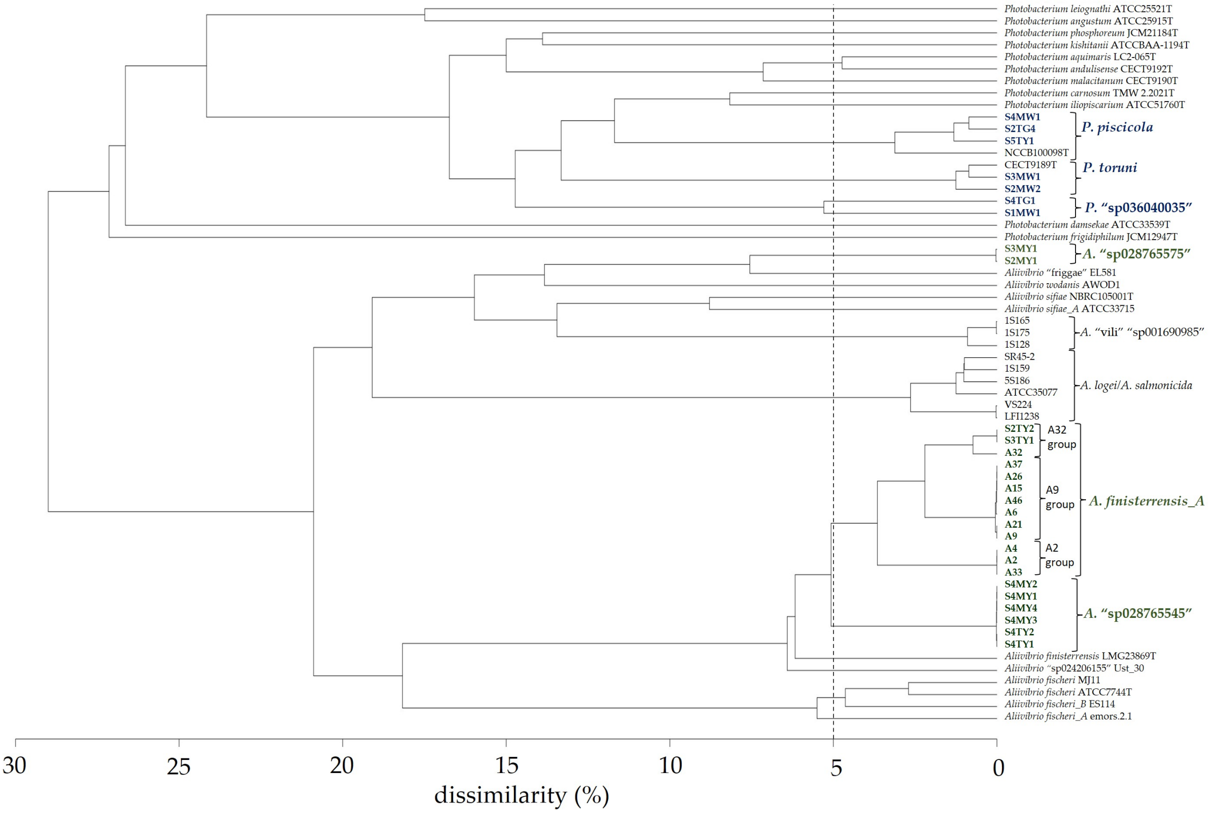

Figure S7. Cladogram based on similarities between *Aliivibrio* strain genomes. The similarities were generated with OrthoANI. The dashed line indicates the notional demarcation point for species discrimination for ANI. Species placeholder designations come from GTDB or Klemetsen et al. (2021).

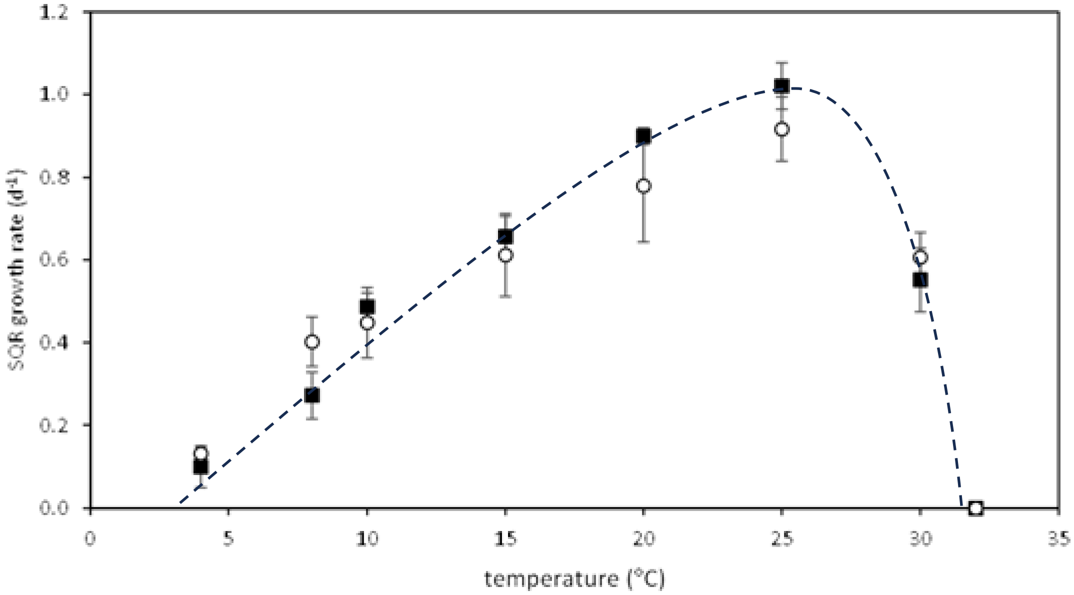

Figure S8. Growth rates over the temperature range for *Aliivibrio* salmon isolates. Average growth rates are determined from multiple strains representing the A2, A9, and A32 groups (n=10). Black squares indicates growth in marine broth while open circles is for growth for marine broth containing 1% (w/v) ox bile salts no. 3. The data distribution is fitted with the Ratkowsky model. Notional T_{min} is 4.2°C and T_{max} 31.8°C. T_{opt} is estimated to be 25.5°C

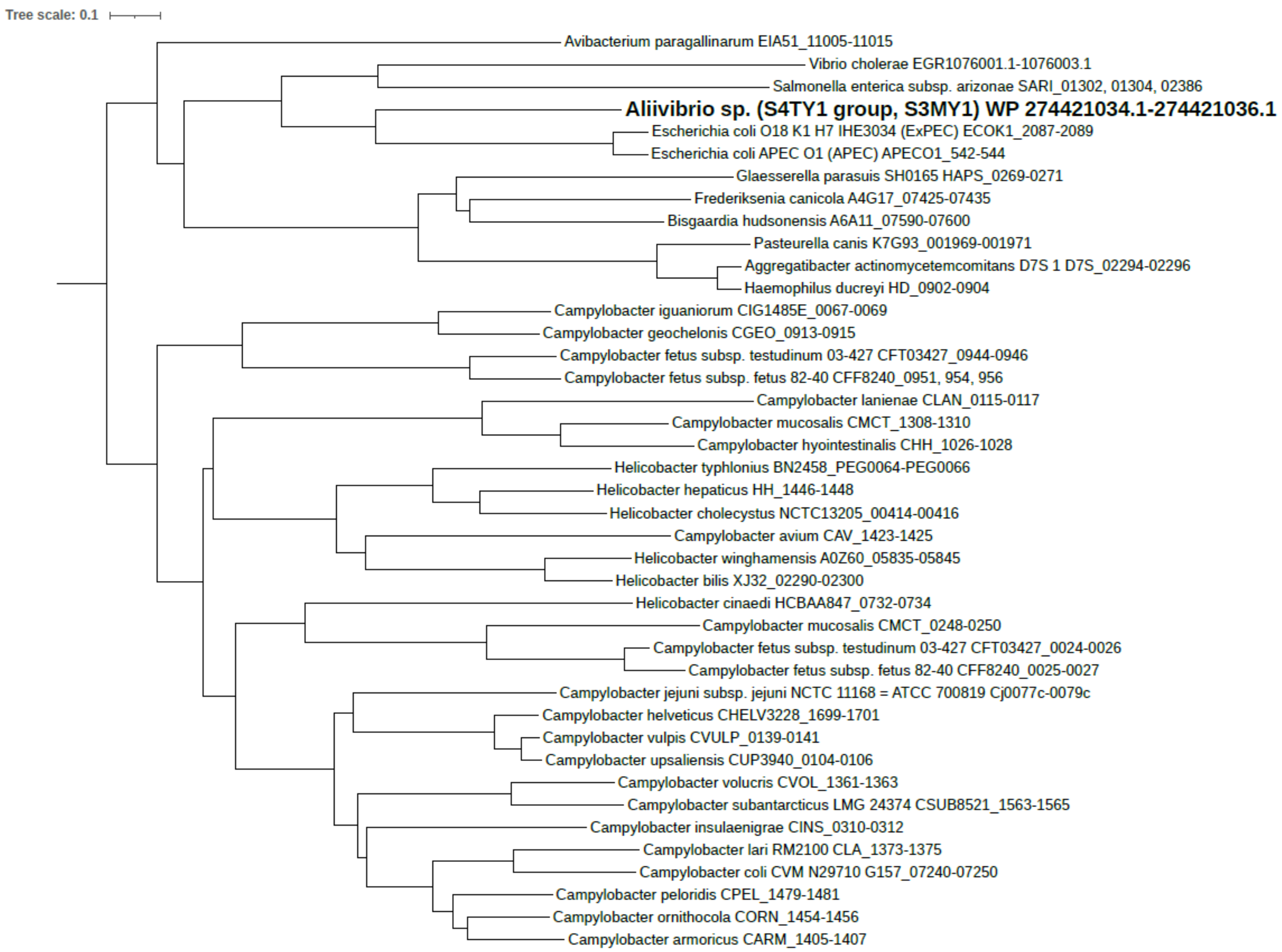

Figure S9. Group average-based tree based on concatenated cytolethal toxin subunit CdtA, CdtB and CdtC protein sequences. Only bacteria that included an intact operon are shown (most taxa shown are annotated in detail in the KEGG database). The CdtABC homologs present in *Aliivibrio* strain S3MY1 and strains of the S4TY1 group had identical amino acid sequences. Closest matches (55% identity overall) was to proteins of pathogenic *E. coli* strains (ExPEC – extraintestinal pathogenic E. coli, APEC – avian pathogenic E. coli). The Cdt sequences from a human faecal *Vibrio cholerae* isolate (no. 633012) deposited into NCBI by the England Public Health in 2020 (PM Ashton and colleagues) was more distantly related but in the same cluster. The expression, function and impact of Cdt in Atlantic salmon is currently unknown. Nor is it know the frequency of *cdt* genes in *Aliivibrio* or other fish-associated bacteria.
